# Quality of Digital Health Interventions Across Different Health Care Domains: Secondary Data Analysis Study

**DOI:** 10.2196/47043

**Published:** 2023-11-23

**Authors:** Maciej Hyzy, Raymond Bond, Maurice Mulvenna, Lu Bai, Alan Dix, Robert Daly, Anna-Lena Frey, Simon Leigh

**Affiliations:** 1 School of Computing Ulster University Belfast United Kingdom; 2 School of Electronics, Electrical Engineering and Computer Science Queen's University Belfast Belfast United Kingdom; 3 Swansea University Swansea United Kingdom; 4 Organisation for the Review of Care and Health Apps (ORCHA) Daresbury United Kingdom

**Keywords:** digital health interventions scoring, digital health interventions, digital health, mHealth assessment, mobile health, ORCHA assessment, Organisation for the Review of Care and Health Apps, quality assessment, quantifying DHIs

## Abstract

**Background:**

There are more than 350,000 digital health interventions (DHIs) in the app stores. To ensure that they are effective and safe to use, they should be assessed for compliance with best practice standards.

**Objective:**

The objective of this paper was to examine and compare the compliance of DHIs with best practice standards and adherence to user experience (UX), professional and clinical assurance (PCA), and data privacy (DP).

**Methods:**

We collected assessment data from 1574 DHIs using the Organisation for the Review of Care and Health Apps Baseline Review (OBR) assessment tool. As part of the assessment, each DHI received a score out of 100 for each of the abovementioned areas (ie, UX, PCA, and DP). These 3 OBR scores are combined to make up the overall ORCHA score (a proxy for quality). Inferential statistics, probability distributions, Kruskal-Wallis, Wilcoxon rank sum test, Cliff delta, and Dunn tests were used to conduct the data analysis.

**Results:**

We found that 57.3% (902/1574) of the DHIs had an Organisation for the Review of Care and Health Apps (ORCHA) score below the threshold of 65. The overall median OBR score (ORCHA score) for all DHIs was 61.5 (IQR 51.0-73.0) out of 100. A total of 46.2% (12/26) of DHI’s health care domains had a median equal to or above the ORCHA threshold score of 65. For the 3 assessment areas (UX, DP, and PCA), DHIs scored the highest for the UX assessment 75.2 (IQR 70.0-79.6), followed by DP 65.1 (IQR 55.0-73.4) and PCA 49.6 (IQR 31.9-76.1). UX scores had the least variance (SD 13.9), while PCA scores had the most (SD 24.8). Respiratory and urology DHIs were consistently highly ranked in the National Institute for Health and Care Excellence Evidence Standards Framework tiers B and C based on their ORCHA score.

**Conclusions:**

There is a high level of variability in the ORCHA scores of DHIs across different health care domains. This suggests that there is an urgent need to improve compliance with best practices in some health care areas. Possible explanations for the observed differences might include varied market maturity and commercial interests within the different health care domains. More investment to support the development of higher-quality DHIs in areas such as ophthalmology, allergy, women’s health, sexual health, and dental care may be needed.

## Introduction

According to a report from 2021 [1], there were more than 350,000 digital health interventions (DHIs) available in the app stores. And in 2020, more than 91,000 DHIs had been added to app stores, which amounts to 251 DHIs per day (on average). Moreover, searches for DHIs within app stores have also increased [2]. A potential catalyst for this could have been the COVID-19 pandemic and restricted access to incumbent services. Nevertheless, these findings clearly indicate that the public has a great interest in the use of DHIs.

However, some of these DHIs may contain harmful content. For example, a study from 2016 [3] conducted a systematic assessment of suicide prevention and deliberate self-harm mobile apps. The study found that some of the apps encouraged risky behaviors, such as the uptake of drugs. Similarly, reviews across different health care domains have demonstrated that many DHIs raise safety, security, or data privacy (DP) concerns [4-8], include incomplete or misleading medical information [9,10], or have not been supported by sufficient scientific evidence [5,6,11]. This indicates that the assessment of DHIs for adherence to best practice standards is critical to ensuring user safety and DHI’s effectiveness, as well as allowing health care professionals to confidently recommend DHIs to clients or patients.

Previous systematic reviews have shown that there are numerous existing assessments for DHI evaluation by experts and users that encompass a large number of heterogenous assessment criteria [12-17]. However, many assessment frameworks demonstrate shortcomings, such as being limited to DHIs in a particular health care domain [12], not including important assessment areas such as DP [12], or being focused on health care professionals without providing meaningful insights to end users [18]. Assessment that addresses these issues, including disease-independent criteria across key areas and making assessment results easily accessible to end users, is needed.

The Organisation for the Review of Care and Health Apps (ORCHA) [19] is a UK-based digital health compliance company that specializes in the assessment of DHI quality in terms of compliance with best practice standards. The Organisation for the Review of Care and Health Apps Baseline Review (OBR) [20] provided by ORCHA is a proxy for DHI’s compliance with best practice standards. ORCHA is currently working with 70% of National Health Service (NHS) organizations within England and provides DHI libraries, hosted by various health care organizations, that contain information about DHIs that have been assessed with the OBR. Specifically, the OBR results provide information (including an assessment score between 0 and 100) regarding DHIs’ compliance with best practices in the domains of professional and clinical assurance (PCA), DP, and user experience (UX), allowing end users and clinical professionals to make informed decisions on whether to use or recommend these DHIs.

Notably, the OBR has been applied to thousands of DHIs, which provides a valuable data set for the investigation of best practice compliance across different types of DHIs. For instance, compliance may vary between DHIs in different health care domains and with different levels of risk (eg, as per the National Institute for Health and Care Excellence [NICE] Evidence Standard Framework [ESF] tier classification [21]). Gaining insights into such variations is important to determine what factors may drive high or low compliance with best practices and which health care domains require more effort and investment to improve the quality of DHIs. Moreover, an understanding of how compliance varies among different types of DHIs can serve as a future reference point for determining how particular DHIs compare to other similar DHIs.

This study aimed to explore these questions using a data set comprising OBR assessment results for 1574 DHIs. In this study, we explore OBR scores regarding 3 NICE tiers and compare the quality of DHIs across 26 different health care domains. We do this by establishing quantiles for each tier and for each health care domain. We want to determine if OBR scores are different across DHIs in different health care domains. This will allow us to identify health care domains that may require more investment to support their development. We hypothesize that the quality of DHIs is different across several health care domains.

## Methods

### The Data Set and Assessment

For this study, ORCHA provided a data set comprising raw data from 1574 DHIs, which were assessed using the OBR version 6 tool [20]. The OBR version 6 is the latest version of the “ORCHA assessment tool,” which consists of almost 300 objective (mostly dichotomous “yes” or “no”) assessment questions in 3 areas: PCA, DP, and UX. Each of the areas is scored individually on a scale from 0 to 100 and combined into an overall ORCHA score.

NICE tiers classify DHIs based on their functionality, risk, and regulatory status. Tier A indicates that the DHIs provide health and social care services with no measurable user outcome. Tier B denotes that the DHIs can provide 2-way communication between users and health care professionals and provide health care information or a health diary. Tier C indicates that DHIs provide preventative behavioral change aimed at health issues; they may allow users to self-manage a specific condition, indicate that DHIs provide or guide treatment for a condition, record and transmit data about this condition to a professional, caregiver, or third party without a user’s input, contain a calculator that impacts treatment, provide diagnostics for a specific condition, or guide a diagnosis [21]. Since the NICE tiers are dependent upon functionality, risk, and regulatory status, it would be inappropriate to, for example, use the OBR score on a tier C DHI using the OBR tier B scoring.

An ORCHA threshold score of 65 is an NHS-accepted cutoff point that indicates compliance with best practice standards for DHIs, meaning that the DHI may be used or recommended by NHS staff. The score of 65 was established with NHS partners in 2020 and has since remained there. It represents the point at which (in the majority), excess risks are avoided; that is, you cannot possibly score above 65 while having no privacy policy, having no relevant evidence, or being a medical device that is not certified.

An ORCHA score of 65 is also an initial score for all the DHIs being assessed in all assessment areas (UX, PCA, and DP). Meaning that the initial score at the beginning of the assessment is 65 for each assessment area and overall ORCHA score. Then, based on answers to assessment questions, this score is altered through value and risk points (value points increase the score and risk points reduce the score) and assigned to a DHI. This process changes the initial score of 65 for each assessment area and is then combined to give an overall ORCHA score. For example, for apps that store personal or sensitive information, value points are assigned to such an app if they make their privacy policy immediately available when the user first uses the DHI. And risk points are assigned if a privacy policy is not clearly available when using the DHI. The amount of value and risk points assigned per question vary based on the NICE ESF tier that has been assigned to a DHI. If no value or risk points were assigned during the assessment, then the ORCHA score remains 65 [20]. Furthermore, to receive full points for appropriate evidence for its ESF tier, a tier B DHI (depending on its exact functionality) may only require a user benefits statement (eg, based on pilot results) and validation of the provided information by experts or references, while a tier C DHI will likely require a full-scale observational study or randomized controlled trial to meet the same evidence threshold. These differences in evidence requirements were introduced by the NICE ESF and adopted with slight amendments by the ORCHA assessment to ensure that standards are realistic and achievable for DHI companies without placing an undue burden on developers of low-risk DHIs, while at the same time setting expectations sufficiently high (especially for high-risk DHIs) to ensure safety and effectiveness and to provide users and health care providers with confidence in the DHIs. Some questions in the ORCHA assessment tool do not assign value or risk points but are there to provide information or context; for example, the question “When was the last Care Quality Commission [22] inspection completed?” does not assign value or risk points.

Each assessment of the 1574 apps has been carried out by at least 2 trained ORCHA reviewers as part of “business as usual” for ORCHA, where in the case of a dispute, a third ORCHA reviewer would resolve it during a discussion. All ORCHA reviewers have undergone the same training to use the OBR version 6 assessment tool.

It takes around 6 months for an ORCHA reviewer to be trained on how to use the OBR and considered ready to carry out live reviews using the tool. The training involves teaching the new reviewer about each area (UX, PCA, and DP) of the OBR. Training is carried out either in person or through web-based meetings.

The data set used included DHI assessments that were published between January 18, 2021, and January 6, 2022. All DHIs were assigned to 26 different health care domains and to 1 of the 3 NICE tiers, established by the NICE ESF [21].

### Statistical Analysis

We carried out secondary data analyses of an ORCHA data set, which comprised the assessment of 1574 DHIs. The data analysis was carried out using R Studio (The R Foundation) and the R programming language (R Core Team). Descriptive statistics, including the minimum score, first quantile, median, mean (SD), third quantile, maximum score, and SE of the mean, were calculated for each of the OBR scores (ORCHA, PCA, DP, and UX).

Box plots were generated to study each score per NICE tier. DHIs were also grouped and analyzed across the different health care domains, with the sample size (number of DHIs) for each health care domain presented. Each OBR score (ORCHA, PCA, DP, and UX) per health care domain has been presented in quantiles from 0% to 100% in increments of 25%. Quantiles have been used so that an easy comparison could be made between different scores, NICE ESF tiers, and health care domains. Normality testing (the Shapiro-Wilk test [23]) was used to determine which hypothesis test was appropriate. The Kruskal-Wallis rank sum test [24] was used to compare the scores between the different NICE tiers, with a *P*<.05 considered statistically significant. The Kruskal-Wallis rank sum test was used to compare the scores across the health care domains with post hoc analysis using the Dunn test [25] and Holm’s [26] method for *P* value adjustment for multiple pairwise comparisons. A 2-sided unpaired Wilcoxon rank sum test has been used to determine if DHIs’ with International Organization for Standardization (ISO) 27001 certification [27] are statistically different from those without, regarding DP scores. The Wilcoxon rank sum test was also used to determine if DHIs classified as medical devices [28] are statistically significantly different than those that are not medical devices, regarding PCA scores. After the Wilcoxon rank sum test, Cliff delta has been used to indicate the magnitude of the difference between 2 compared samples of DHIs with a 95% CI. Cliff delta magnitude has been assessed using the thresholds |d|<.147 “negligible,” |d|<.33 “small,” |d|<.474 “medium,” otherwise “large” [29]. The above analyses have been conducted for all DHIs, separated by NICE tiers (n=number of DHIs), tier B (n=1155), and tier C (n=408). Tier A (n=11) was excluded due to sample size.

### Ethical Considerations

This secondary data analysis study gained ethical approval (project number: CEBE_RE-22-002) by Ulster University (ethics filter committee, Faculty of Computing, Engineering, and the Built Environment). The process undertaken by ORCHA ensures that DHIs’ developers are aware of their score and are given time to contest the findings of the assessment, which may be amended if developers provide additional relevant information. All reviews, unless explicitly asked to be removed by the developer, are covered as suitable for research in ORCHA’s privacy policy [30].

## Results

### Overview

**Figure 1:**
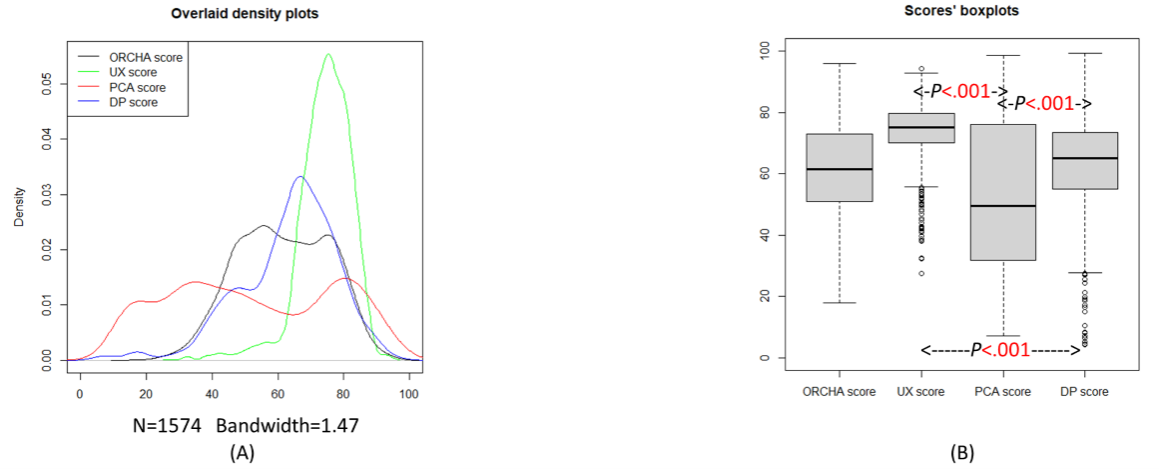
(A) Overlaid density plot of the Organisation for the Review of Care and Health Apps (ORCHA) score, professional and clinical assurance (PCA) score, data privacy (DP) score, and user experience (UX) score for 1574 digital health interventions (DHIs). (B) Box plots of scores for 1574 DHIs. The Kruskal-Wallis test had *P*<.001, and the posthoc Dunn test on score distributions for UX, PCA, and DP, all had *P*<.001.

Table 1 presents a summary of the OBR scores for all DHIs. A Kruskal-Wallis test revealed that the distributions of the UX, PCA, and DP scores were statistically significantly different from each other (*P*<.001). Figure 1A shows that UX scores have the least variance of the 3 assessment areas, whereas PCA scores have the greatest variance. Table 1 shows that the SD for UX scores is 8.20, whereas the SD for PCA scores is 24.8, which is approximately 3 times greater than the SD of the UX scores. The UX scores are also typically higher than the other scores. A total of 57.3% (902/1574) of DHIs in the data set have an ORCHA score below the accepted ORCHA threshold of 65. Multimedia Appendix 1 contains the number of DHIs with varied ORCHA thresholds. Multimedia Appendix 2 contains the steps involved in selecting 1574 DHIs.

**Table 1:** Summary of scores for the 1574 digital health interventions (DHIs).

Score	0%	25%	50%	75%	100%	Mean (SD)	SEM^a^
ORCHA^b^	18.0	51.0	61.5	73.0	96.0	61.6 (13.9)	.350
UX^c^	27.4	70.0	75.2	79.6	94.2	74.3 (8.20)	.207
PCA^d^	7.14	31.9	49.6	76.1	98.5	52.2 (24.8)	.626
DP^e^	4.28	55.0	65.1	73.4	99.3	63.2 (14.9)	.375

^a^SEM: standard error of the mean.

^b^ORCHA: Organisation for the Review of Care and Health Apps.

^c^UX: user experience.

^d^PCA: professional and clinical assurance.

^e^DP: digital privacy.

Table 1 shows the quantiles for each of the scores from 0% to 100% in increments of 25%. The ORCHA score for the 50% (median) quantile is 61.5, which is below ORCHA’s threshold score of 65, meaning that most of the DHIs in the data set fail to adhere to the NHS cutoff for compliance with best practice standards. Median (IQR) for the OBR scores are ORCHA (61.5, IQR 51.0-73.0), UX (75.2, IQR 70.0-79.6), PCA (49.6, IQR 31.9-76.1), and DP (65.1, IQR 55.0-73.4).

### Scores per NICE Tier

**Figure 2:**
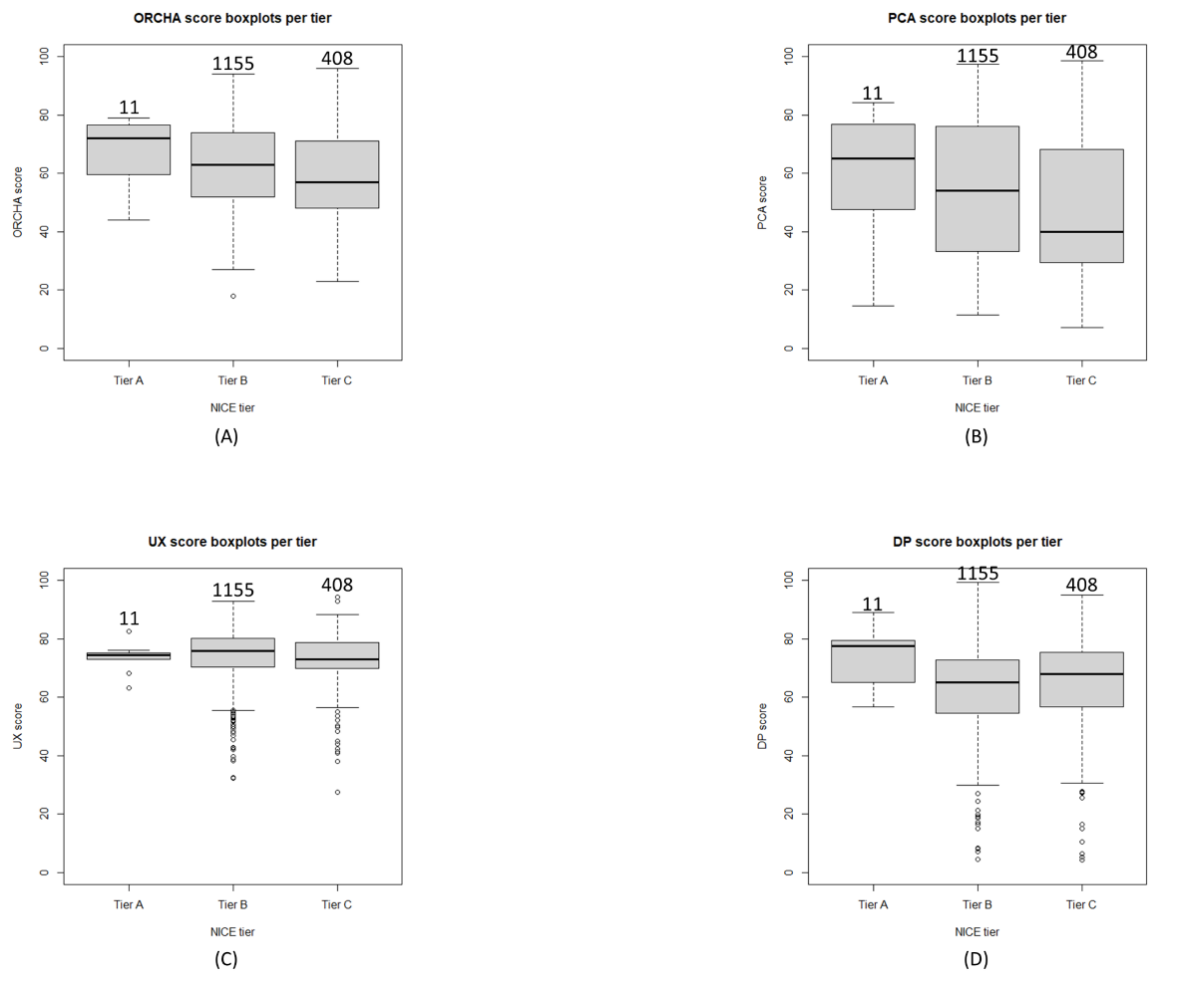
The number above the box plots is the sample size. (A) Organisation for the Review of Care and Health Apps (ORCHA) score box plots per National Institute for Health and Care Excellence (NICE) tier. (B) Professional and clinical assurance (PCA) score box plots per NICE tier. (C) User experience (UX) score box plots per NICE tier. (D) Data privacy (DP) score box plots per NICE tier.

DHIs were distributed as follows across the different NICE tiers: tier A (11/1574, 0.699%), tier B (1155/1574, 73.4%), and tier C (408/1574, 25.9%). Figure 2 depicts box plots for the OBR scores within each tier. Further information is provided in Multimedia Appendix 3, which depicts quantiles for each score and NICE tier permutations from 0% to 100% in increments of 25%.

### Scores by Health Care Domains

**Figure 3:**
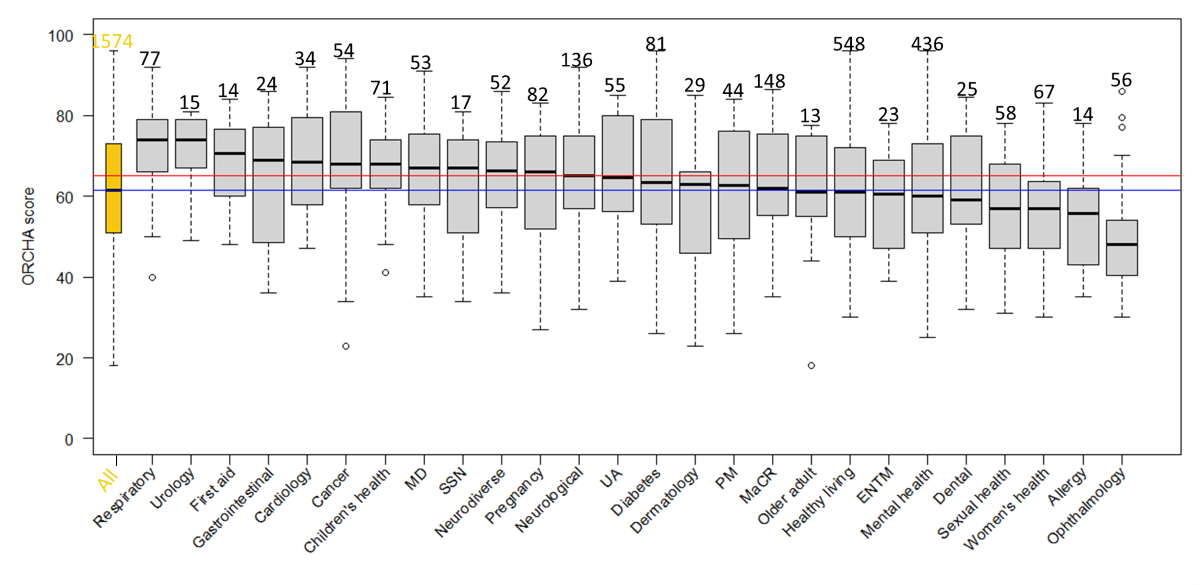
The 1574 digital health interventions (DHIs) are classified into categories (can be more than one), an overall Organisation for the Review of Care and Health Apps (ORCHA) score box plot (first from left), and ORCHA scores box plots per health care domain. Sample sizes are above box plots; the red line indicates an ORCHA threshold score of 65, and the blue line indicates an overall median score of 61.5. The Kruskal-Wallis rank sum test has *P*<.001. ENTM: ear, nose, throat, and mouth; MaCR: medicines and clinical reference; MD: musculoskeletal disorders; PM: pain management; SSN: social support network; UA: utilities or administration.

**Figure 4:**
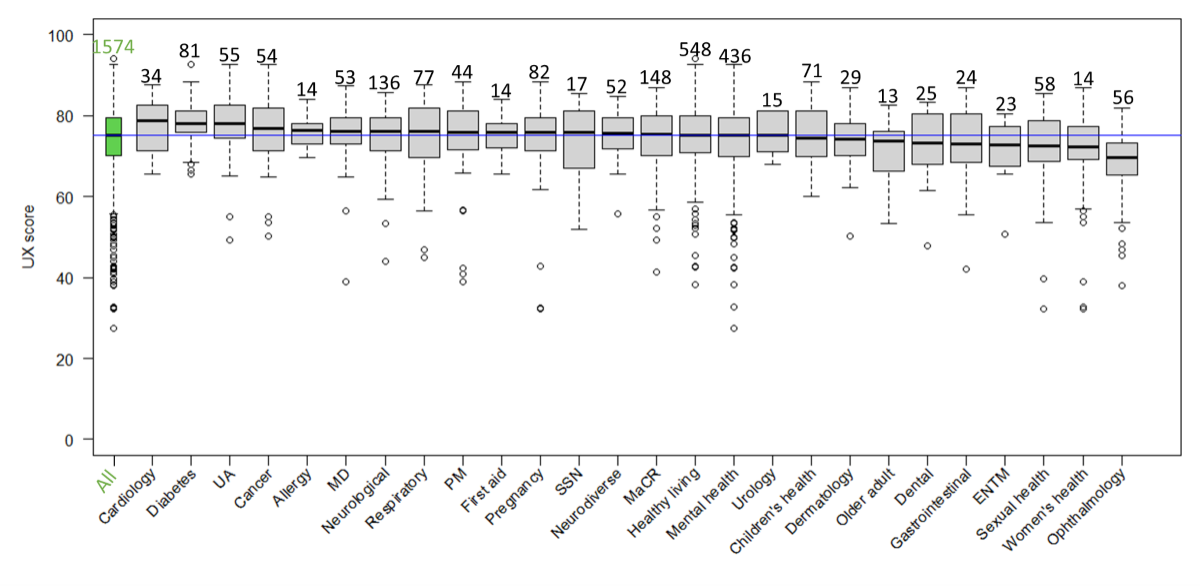
The 1574 digital health interventions (DHIs) are classified into categories (can be more than 1), an overall user experience (UX) score box plot (first from left), and UX scores box plots per health care domain. Sample sizes are above box plots; the blue line indicates an overall median score of 75.2. The Kruskal-Wallis rank sum test has *P*<.001. ENTM: ear, nose, throat, and mouth; MaCR: medicines and clinical reference; MD: musculoskeletal disorders; PM: pain management; SSN: social support network; UA: utilities or administration.

**Figure 5:**
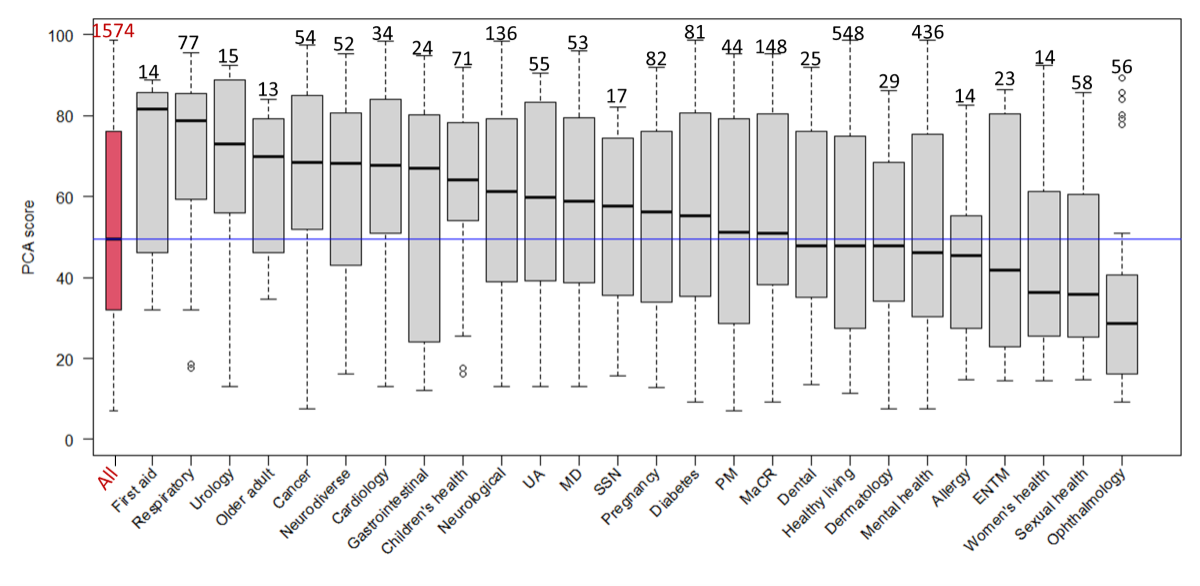
The 1574 digital health interventions (DHIs) are classified into categories (can be more than one), an overall professional and clinical assurance (PCA) score box plot (first from left), and PCA scores box plots per health care domain. Sample sizes are above box plots; the blue line indicates an overall median score of 49.6. The Kruskal-Wallis rank sum test has *P*<.001. ENTM: ear, nose, throat, and mouth; MaCR: medicines and clinical reference; MD: musculoskeletal disorders; PM: pain management; SSN: social support network; UA: utilities or administration.

**Figure 6:**
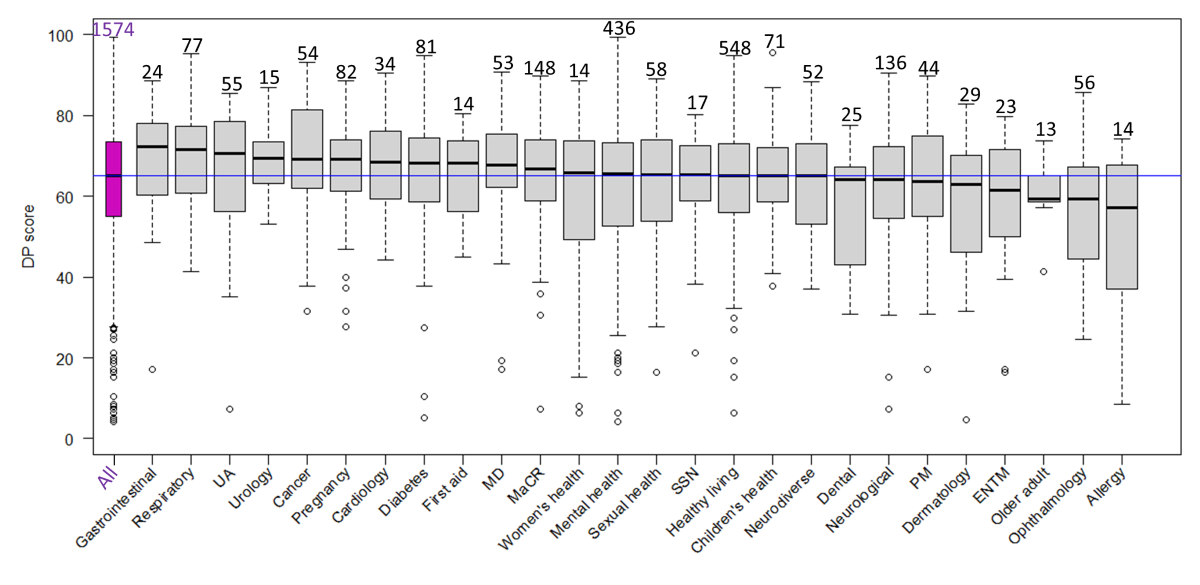
The 1574 digital health interventions (DHIs) are classified into categories (can be more than one), an overall digital privacy (DP) score box plot (first from left), and DP scores box plots per health care domain. Sample sizes are above box plots; the blue line indicates an overall median score of 65.1. The Kruskal-Wallis rank sum test has *P*<.001. ENTM: ear, nose, throat, and mouth; MaCR: medicines and clinical reference; MD: musculoskeletal disorders; PM: pain management; SSN: social support network; UA: utilities or administration.

The highest number of DHIs fell into the health care domains of healthy living (n=548) and mental health (n=436). Multimedia Appendix 4 provides a table of health care domains, including the number of DHIs within each health care domain (ie, the sample size) and the scores’ quantiles from 0% to 100% in increments of 25%. Figures 3-6 show the distribution of scores within each health care domain as box plots in descending order of the median (except for the first box plot that shows overall performance). Further details regarding OBR scores (ORCHA, UX, PCA, and DP) for each health care domain can be found in Multimedia Appendices 4 and 5.

Kruskal-Wallis rank sum tests were used to check for statistically significant differences between DHI categories. A statistically significant result (*P*<.001) was obtained for all OBR scores (ORCHA, UX, PCA, and DP) meaning that for all the scores at least 1 health care domain distribution is statistically significantly different from another. A post hoc analysis was conducted using the Dunn test to identify which categories are statistically different from each other (Multimedia Appendix 6).

For all DHIs, a total of 46.2% (12/26) health care domains had a median ORCHA score of 65 or more. The apps in each of the health care domains presented in descending order of quality (median ORCHA score; n) are as follows: respiratory (median 74.0; n=77), urology (median 74.0; n=15), first aid (median 70.5; n=14), gastrointestinal (median 69.0; n=24), cardiology (median 68.5; n=34), children’s health (median 68.0; n=71), cancer (median 68.0; n=54), social support network (median 67.0; n=17), musculoskeletal disorders (median 67.0; n=53), neurodiverse (median 66.3; n=52), pregnancy (median 66.0; n=82), and neurological (median 65.0; n=136). A total of 53.8% (14/26) health care domains had a median ORCHA score of less than 65. These, in descending order, are as follows: utilities or administration (median 64.5; n=55); diabetes (median 63.5; n=81); dermatology (median 63.0; n=29); pain management (median 62.8; n=44); medicines and clinical reference (median 62.0; n=148); healthy living (median 61.0; n=548); older adult (median 61.0; n=13); mental health (median 60.0; n=436); ear, nose, throat, and mouth (median 60.5; n=23); dental care (median 59.0; n=25); women’s health (median 57.0; n=67); sexual health (median 57.0; n=58); allergy (median 55.8; n=14); and ophthalmology (median 48.0; n=56).

For tier B, a total of 57.7% (15/26) health care domains had a median ORCHA score of 65 or more. These, in descending order, are as follows: cancer (median 75.0; n=37), respiratory (median 73.0; n=49), urology (median 71.5; n=12), pregnancy (median 70.8; n=56), first aid (median 70.5; n=14), utilities or administration (median 70.0; n=41), children’s health (median 68.0; n=66), social support network (median 68.0; n=16), neurological (median 68.0; n=104), medicines and clinical reference (median 68.0; n=95), neurodiverse (median 67.5; n=48), diabetes (median 67.5; n=33), musculoskeletal disorders (median 67.0; n=30), older adult (median 66.5; n=10), and cardiology (median 66.0; n=16). A total of 42.3% (11/26) health care domains had a median ORCHA score of less than 65. These, in descending order, are as follows: dermatology (median 64.3; n=16); pain management (median 63.5; n=35); mental health (median 62.0; n=332); sexual health (median 62.0; n=27); healthy living (median 61.0; n=436); dental care (median 59.5; n=20); women’s health (median 58.3; n=36); allergy (median 58.0; n=9); gastrointestinal (median 56.0; n=13); ear, nose, throat, and mouth (median 55.0; n=17); and ophthalmology (median 50.0; n=30).

For tier C, a total of 24% (6/25; no “first aid” health care domain) health care domains had a median ORCHA score of 65 or more. These, in descending order, are as follows: urology (median 79.0; n=3), respiratory (median 74.5; n=28), cardiology (median 72.5; n=18), gastrointestinal (median 71.0; n=11), children’s health (median 70.0; n=5), and musculoskeletal disorders (median 68.0; n=23). A total of 76% (19/25) health care domains had a median ORCHA score of less than 65. These, in descending order, are as follows: cancer (median 64.0; n=17); diabetes (median 63.0; n=48); ear, nose, throat, and mouth (median 61.8; n=6); healthy living (median 60.0; n=106); pain management (median 59.0; n=9); women’s health (median 57.0; n=31); neurological (median 57.0; n=30); utilities or administration (median 57.0; n=14); medicines and clinical reference (median 55.5; n=49); mental health (median 55.0; n =102); pregnancy (median 55.0; n=26); older adult (median 55.0; n=2); sexual health (median 51.0; n=31); dermatology (median 46.0; n=13); neurodiverse (median 46.0; n=3); dental care (median 44.0; n=5); ophthalmology (median 43.3; n=26); allergy (median 42.0; n=5); and social support network (median 34.0; n=1). Multimedia Appendix 5 contains UX, PCA, and DP assessment areas ranked in order, and Multimedia Appendix 7 contains rank consistency. Multimedia Appendix 8 contains Distribution of DHIs across NICE Evidence Standards Framework (ESF) tiers by healthcare domain.

### Partition of DHIs by ISO Certification and Medical Device Designation

Using median (IQR), the following difference has been found in DP scores among DHIs that received ISO 27001 certification [27] (79.4, IQR 73.6-85.3; n=77) and those that did not (65.0, IQR 54.1-72.4; n=1497), with a 2-sided unpaired Wilcoxon rank sum test with *P*<.001 and Cliff delta =.704 (95% CI 0.620-0.772). The following difference has been found in PCA scores among DHIs that have been designated as “medical device” [28] (58.8, IQR 33.7-84.4; n=162) and those that were not (49.3, IQR 31.9-76.1; n=1412), with a 2-sided unpaired Wilcoxon rank sum test with *P*=.003 and Cliff delta =.143 (95% CI 0.040-0.243).

For tier B, the following difference has been found in DP scores among DHIs that received ISO 27001 certification (78.5, IQR 71.4-81.8; n=42) and those that did not (65.0, IQR 53.8-72.2; n=1113), with a 2-sided unpaired Wilcoxon rank sum test with *P*<.001 and Cliff delta =.667 (95% CI 0.541-0.764). The following difference has been found in PCA scores among DHIs that have been designated as “medical device” (78.3, IQR 41.8-86.7; n=23) and those that were not (50.9, IQR 31.9-76.1; n=1132), with a 2-sided unpaired Wilcoxon rank sum test with *P*<.001 and Cliff delta =.644 (95% CI 0.470-0.769).

For tier C, the following difference has been found in DP scores among DHIs that received ISO 27001 certification (83.2, IQR 75.7-86.4; n=35) and those that did not (66.8, IQR 54.1-73.6; n=373), with a 2-sided unpaired Wilcoxon rank sum test with *P*<.001 and Cliff delta =.724 (95% CI 0.604-0.812). The following difference has been found in PCA scores among DHIs that have been designated as “medical device” (43.7, IQR 28.7-80.8; n=139) and those that were not (41.1, IQR 30.3-68.2; n=269), with a 2-sided unpaired Wilcoxon rank sum test with *P*=.002 and Cliff delta =.183 (95% CI 0.061-0.300).

## Discussion

### Principal Findings

A total of 57.3% (902/1574) DHIs in the data set failed to meet the ORCHA (a proxy for overall quality) threshold score of 65. The UX score was consistently the highest out of the 3 assessment areas (UX, PCA, and DP). The UX score also had the least variance when compared with other OBR scores. We found that scores differed widely between different health care domains. However, only some differences achieved statistical significance (Dunn test in Multimedia Appendix 6). The analysis revealed that the highest ORCHA scores were observed in the respiratory health care domain and the lowest in the ophthalmology health care domain (Figure 1 and Multimedia Appendix 4).

There have been several studies that suggest DHIs’ quality could be further improved [4-8]. By identifying health care domains that have low OBR DHIs, this study indicates where a greater effort is needed to quality-assure these DHIs.

Table 1 shows that the largest variance has been observed in the PCA assessment area (PCA score IQR 44.2 and SD 24.8), which includes criteria related to the availability of scientific evidence to support the content and efficacy or effectiveness of the DHIs. This variation in clinical assurance across different DHIs is consistent with previous research. For instance, a paper from 2021 [31] found that evidence to support the claims made by health apps is often unavailable or of questionable quality. Similarly, a systematic review and exploratory meta-analysis from 2017 [32] with a focus on diagnostic apps found that the evidence for the diagnostic performance of health apps is limited. Additionally, a meta-analysis of randomized controlled trials from 2021 [27] concluded that, while there has been an increase in the rigorous evaluation of apps aimed at modifying behavior to promote health and manage disease, the evidence that such apps can improve health outcomes is weak.

Previous work has been done on benchmarking DHI system usability scores (SUS) across digital health apps [33] and for heart failure apps [34]. This study differs as it focuses on comparing DHIs using a broader selection of DHIs across health care domains and assessment areas (UX, PCA, and DP). Previous work from 2020 [35] has introduced an implementation framework called Technology Evaluation and Assessment Criteria for Health Apps. The aim of the framework is to enable users to make informed decisions regarding app use and increase app evaluation engagement by introducing a process to assist app implementation (Technology Evaluation and Assessment Criteria for Health Apps) across all DHIs. This study differs as it not only enables users to make informed decisions regarding app use but also enables the comparison of DHIs across health care domains. This study also identifies which health care domains may need more attention regarding their quality.

### Compliance With Best Practices Across Health Care Domains

This study further observed differences in best practice compliance among health care domains. While DP and UX median scores were relatively similar across health care domains, large differences were observed between PCA scores (Figure 5 and Multimedia Appendices 4 and 5). A potential partial explanation for these findings may be that the proportion of DHIs within different tiers, and thus with different levels of evidence requirements (see above), may vary among health care domains. This suggestion is partially supported by the data, as a large proportion of DHIs in health care domains with high PCA scores fall into tiers A or B rather than C (Multimedia Appendices 5 and 8).

For all DHIs, a total of 12 of the 26 health care domains had a median ORCHA score of 65 or more. And a total of 14 of the 26 health care domains had a median ORCHA score of less than 65. For tier B, a total of 15 of the 26 health care domains had a median ORCHA score of 65 or more. And 11 of the 26 health care domains had a median ORCHA score of less than 65. For tier C, a total of 6 of the 25 (no “first aid” health care domain) health care domains had a median ORCHA score of 65 or more. And 19 of the 25 health care domains had a median ORCHA score of less than 65. Respiratory and urology DHIs were consistently highly ranked in NICE tiers B and C (Multimedia Appendices 4, 5, and 7).

The data indicate that DHIs that have received ISO 27001 certification (median 79.4, IQR 73.6-85.3; n=77) score higher regarding their DP score than those that have not (median 65.0, IQR 54.1-72.4; n=1497). The difference was statistically significant with a Wilcoxon rank sum test with *P*<.001 and Cliff delta =.704, indicating a large difference in DP scores. Similar results were obtained when the DHIs were partitioned by tiers B and C, as can be seen in the “Partition of DHIs by ISO Certification and Medical Device Designation” section.

DHIs that have been designated as medical device (median 58.8, IQR 33.7-84.4; n=162), scored higher on PCA than those that were not (median 49.3, IQR 31.9-76.1; n=1412). The difference was statistically significant with a Wilcoxon rank sum test with *P*=.003 and Cliff delta=.143, indicating a negligible difference in PCA scores. However, when partitioned by NICE tiers B and C, as can be seen in the “Partition of DHIs by ISO Certification and Medical Device Designation” section, results showed that for tier B DHIs that have been designated as medical device (median 78.3, IQR 41.8-86.7; n=23) scored higher than those that were not (median 50.9, IQR 31.9-76.1; n=1132). And had a Wilcoxon rank sum test with *P*<.001 and Cliff delta =.644, indicating a large difference in PCA scores. For tier C, DHIs that have been designated as medical device (median 43.7, IQR 28.7-80.8; n=139) and those that were not (median 41.1, IQR 30.3-68.2; n=269) had a Wilcoxon rank sum test with *P*=.002, but a much lower Cliff delta =.183, indicating a negligible difference in PCA scores.

Medical device DHIs seem to be outperforming nonmedical device DHIs regarding PCA scores. Especially medical device DHIs in tier B. Speculation can be made that since medical device DHIs have regulatory requirements [28], more is expected of them regarding PCA. This leads to low PCA scores among tier C apps, with a negligible difference in PCA score between medical device and nonmedical device DHIs, according to Cliff delta. However, since medical device DHIs are typically assigned to tier C, they outperform nonmedical device DHIs in tier B as developers attempt to meet regulatory demands. An alternative interpretation is that since medical device regulation is a gold standard where clinical evidence is evaluated, it would be expected to see higher PCA scores for DHIs designated as medical devices than nonmedical devices in tier C, similarly to DHIs in tier B. It could be that the PCA score for tier C is an inappropriate measure of clinical evidence. Meaning that the criteria for tier C DHIs are not ideal to differentiate between different levels of evidence.

A study from 2020 [36] focused on the value of mobile health (mHealth) for patients. Their analysis found that the highest level of clinical evidence for mHealth apps used for clinical scenarios is scarce. The analysis presented in this study identifies health care domains where DHIs may require improvements regarding their quality. Hence, this study may be helpful in mitigating the problem of scarce evidence regarding the quality of DHIs.

The current findings indicate that OBR scores differ among DHIs in different NICE tiers and health care domains. In the long term, the aim should be to elevate DHIs in lower-scoring categories to achieve an ORCHA threshold score of 65. The quantiles presented in Multimedia Appendices 3 and 4 can be used for the identification of low-quality DHIs as indicated by OBR scores.

After receiving OBR scores, a specific DHI can be compared with other DHIs in the same health care domain or NICE tier using quantiles. This will reveal how compliant the DHI is with best practice standards relative to similar DHIs. These comparisons can be conducted with ORCHA scores or for the separate assessment areas (UX, PCA, and DP; Multimedia Appendices 3 and 4).

### Limitations

A few limitations of this study should be noted. There were uneven sample sizes for DHIs across NICE tiers (the sample size ranged from 11 to 1155 DHIs) and health care domains (the sample size ranged from 13 to 548 DHIs). When partitioning the data, lower samples in tiers and categories lead to less reliable results in those tiers and categories. Where the case was that the same DHIs were assessed twice, that is, the Android version and the iOS version (n=466 DHIs; Multimedia Appendix 2), the mean OBR scores were calculated using the Android and iOS assessments, and the result was included in the analysis. However, it is possible that if the names of the DHIs were somewhat different for the Android and iOS versions, both would have been included in the analysis as separate DHIs.

The OBR version 6 evolved from earlier versions of the OBR during the height of the COVID-19 pandemic. Originally, version 6 was created as a more stringent version of the OBR so that ORCHA could recommend the most compliant DHIs to members of the UK population with confidence. ORCHA tested version 6 on a selection of highly compliant DHIs (as determined by previous versions of the OBR). This set of 30 DHIs served as the pilot group, with the subsequent 2097 DHIs being assessed with ORCHA’s typical assessment approach of categorizing DHIs into categories, ordering by number of downloads, and assessing the most downloaded DHI in each health care domain, followed by the second, and so forth.

### Future Work

The concurrent validity can be performed on the ORCHA assessment tool by comparing ORCHA scores against other assessment frameworks (eg, Mobile Application Rating Scale [37]). The analysis conducted in this paper could be repeated with more DHIs in tier A.

### Conclusion

This study examined assessment data for 1574 DHIs and found that 57.3% (902/1574) of the DHIs in the data set failed to meet the ORCHA threshold score of 65 (accepted by the NHS as a signal of compliance with best practice standards). This work also identified differences with regard to the OBRs of DHIs in different tiers and health care domains. Appropriate evidence and clinical assurance were especially lacking in DHIs with high risk (as per their tiers), which raises safety concerns and highlights the need for DHI assessments that support users in the selection of safe and effective DHIs. Interestingly, more stringent (tier C) clinical assurance and evidence requirements seemed more likely to be met in health care domains with high funding availability, such as diabetes and cardiology. This underscores the need for more investment in health care domains that currently demonstrate low compliance with best practices, such as women’s health, ophthalmology, dental care, and allergy.

Additionally, this study produced quantiles across different health care domains and NICE tiers, which could be used to compare health care domain-specific DHIs in future studies.

## Appendix

Multimedia Appendix 1 Different ORCHA thresholds.

Multimedia Appendix 2 Inclusion of digital health interventions (DHIs) in the Analysis.

Multimedia Appendix 3 Organisation for the Review of Care and Health Apps Baseline Review (OBR) V6 score quantiles separated by National Institute for Health and Care Excellence (NICE) tiers.

Multimedia Appendix 4 OBR V6 score quantiles classified into healthcare domains.

Multimedia Appendix 5 Rank of DHIs.

Multimedia Appendix 6 Post hoc analysis with Dunn test.

Multimedia Appendix 7 DHI rank consistency.

Multimedia Appendix 8 Distribution of DHIs across NICE Evidence Standards Framework (ESF) tiers by healthcare domain.

## Abbreviations

DHI: digital health intervention
DP: data privacy
ESF: Evidence Standards Framework
ISO: International Organization for Standardization
mHealth: mobile health
NHS: National Health Service
NICE: National Institute for Health and Care Excellence
OBR: Organisation for the Review of Care and Health Apps Baseline Review
ORCHA: Organisation for the Review of Care and Health Apps
PCA: professional and clinical assurance
SUS: system usability scores
UX: user experience

## Data Availability

The data sets generated during and/or analyzed during this study are available in the ORCHA repository [38]. The data from this data set (and for more DHIs) are freely available to registered users (registration is free for a trial period). However, the data in the repository are presented for individual DHIs and would need to be extracted into a suitable format for analysis.
