# Supplementary material for: Quality of Digital Health Interventions Across Different Health Care Domains: Secondary Data Analysis Study
Source: JMIR Mhealth Uhealth. 2023 Nov 23;11:e47043. doi: 10.2196/47043 (PMC10704310; doi:10.2196/47043)
Supplement: Multimedia Appendix 1 [file mhealth_v11i1e47043_app1.docx]

## Appendix 1 – Different ORCHA thresholds

Current ORCHA threshold is 65 (highlighted in the appendix 5 table 1)

**Appendix 5 Table 1:** ORCHA thresholds and number of DHIs below them

| **Threshold** | **No. of DHIs below threshold** | **No. of DHIs below threshold**  **(% out of 1574)** |
| --- | --- | --- |
| 0 | 0 | 0% |
| 5 | 0 | 0% |
| 10 | 0 | 0% |
| 15 | 0 | 0% |
| 20 | 1 | 0.06% |
| 25 | 2 | 0.13% |
| 30 | 9 | 0.57% |
| 35 | 28 | 1.78% |
| 40 | 84 | 5.34% |
| 45 | 167 | 10.61% |
| 50 | 350 | 22.24% |
| 55 | 529 | 33.61% |
| 60 | 722 | 45.87% |
| 65 | 902 | 57.31% |
| 70 | 1064 | 67.60% |
| 75 | 1227 | 77.95% |
| 80 | 1414 | 89.83% |
| 85 | 1522 | 96.70% |
| 90 | 1561 | 99.17% |
| 95 | 1573 | 99.94% |
| 100 | 1574 | 100% |


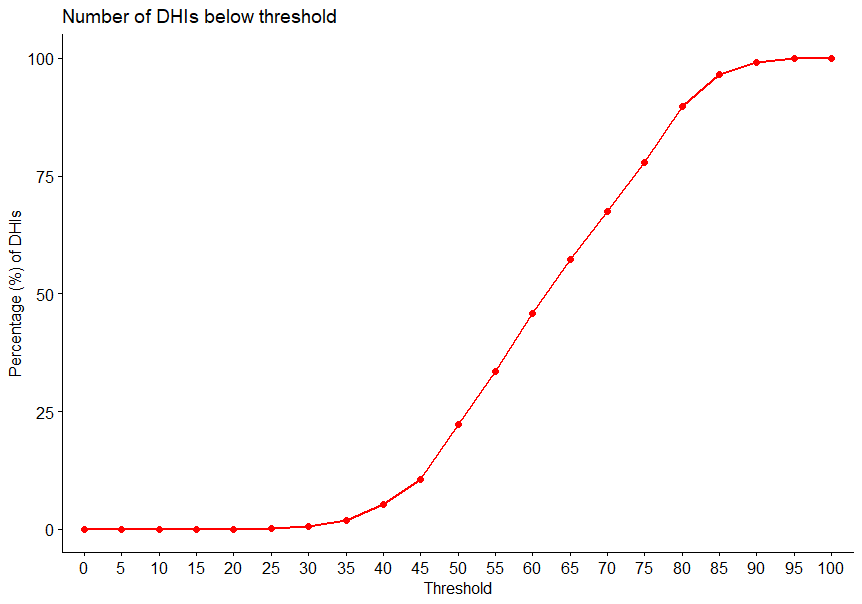


**Appendix 5 Figure 1:** Line graphs showing percentage of apps below each threshold.
