# Supplementary material for: Quality of Digital Health Interventions Across Different Health Care Domains: Secondary Data Analysis Study
Source: JMIR Mhealth Uhealth. 2023 Nov 23;11:e47043. doi: 10.2196/47043 (PMC10704310; doi:10.2196/47043)
Supplement: Multimedia Appendix 2 [file mhealth_v11i1e47043_app2.docx]

## Appendix 2 –Inclusion of DHIs in the Analysis

Appendix 2 Figure 1 shows how the 1574 DHIs have been selected. To avoid inclusion of the same app twice in the analysis, the mean of the scores (ORCHA, PCA, UX and DP) has been taken from the two versions (iOS and Android) and included in the analysis as one DHI.

App assessments in the ORCHA dataset

Dataset (n = 2127)

n= Number of app assessments

Apps assessed for eligibility

(n = 2127)

Apps excluded:

Second assessments (n = 38)

Not a DHI (n = 36)

Healthcare domain sample size <10 (n=13)

Eligible DHIs

(n = 2040)

**Inclusion of DHIs in the Analysis**

DHIs analysed

(n = 1574)

The same DHIs that were assessed twice i.e. the android version and the iOS version. Where this is the case, the mean quality scores were calculated using the Android and iOS assessment:

(n=466)

**Appendix 2 Figure 1:** DHI selection diagram. Second assessment is when the same app has been assessed twice at different times.
