# Supplementary material for: Quality of Digital Health Interventions Across Different Health Care Domains: Secondary Data Analysis Study
Source: JMIR Mhealth Uhealth. 2023 Nov 23;11:e47043. doi: 10.2196/47043 (PMC10704310; doi:10.2196/47043)
Supplement: Multimedia Appendix 3 [file mhealth_v11i1e47043_app3.docx]

Appendix 3 – OBR V6 score quantiles separated by NICE tiers

DHIs samples sizes (n) – tier A: n=11 (.699%), tier B: n=1155 (73.4%), tier C: n=408 (25.9%). Colour code – red for values >75, green for values 65-75 and blue for values <65.

| **Scores** | **NICE tier** | **0%** | **25%** | **50%** | **75%** | **100%** |
| --- | --- | --- | --- | --- | --- | --- |
| ORCHA | Tier A | 44.0 | 59.5 | 72.0 | 76.5 | 79.0 |
|  | Tier B | 18.0 | 52.0 | 63.0 | 74.0 | 94.0 |
|  | Tier C | 23.0 | 48.0 | 57.0 | 71.0 | 96.0 |
| UX | Tier A | 63.2 | 73.0 | 74.4 | 75.2 | 82.6 |
|  | Tier B | 32.2 | 70.3 | 75.8 | 80.2 | 92.7 |
|  | Tier C | 27.4 | 69.8 | 72.9 | 78.8 | 94.2 |
| PCA | Tier A | 14.5 | 47.7 | 65.0 | 76.7 | 84.1 |
|  | Tier B | 11.4 | 33.2 | 54.1 | 76.2 | 97.4 |
|  | Tier C | 7.14 | 29.4 | 40.0 | 68.1 | 98.5 |
| DP | Tier A | 56.8 | 65.0 | 77.6 | 79.3 | 89.0 |
|  | Tier B | 4.62 | 54.4 | 65.0 | 72.6 | 99.3 |
|  | Tier C | 4.28 | 56.6 | 67.8 | 75.3 | 94.9 |
