# Supplementary material for: Quality of Digital Health Interventions Across Different Health Care Domains: Secondary Data Analysis Study
Source: JMIR Mhealth Uhealth. 2023 Nov 23;11:e47043. doi: 10.2196/47043 (PMC10704310; doi:10.2196/47043)
Supplement: Multimedia Appendix 4 [file mhealth_v11i1e47043_app4.docx]

Appendix 4 – OBR V6 score quantiles classified into healthcare domains

Healthcare domains in a descending order of sample size (n). Colour code – red for values >75, green for values 65-75 and blue for values <65.

| **Index** | **Healthcare domains** | **Sample size (n)** | **Scores** | **0%** | **25%** | **50%**  **(median)** | **75%** | **100%** |
| --- | --- | --- | --- | --- | --- | --- | --- | --- |
| 1 | Healthy living | 548 | ORCHA | 30.0 | 50.0 | 61.0 | 72.0 | 96.0 |
|  |  |  | UX | 38.2 | 70.8 | 75.2 | 79.7 | 94.2 |
|  |  |  | PCA | 11.4 | 27.5 | 47.7 | 74.7 | 98.5 |
|  |  |  | DP | 6.34 | 55.9 | 65.0 | 73.0 | 94.9 |
| 2 | Mental health | 436 | ORCHA | 25.0 | 51.0 | 60.0 | 73.0 | 96.0 |
|  |  |  | UX | 27.4 | 69.8 | 75.2 | 79.6 | 92.7 |
|  |  |  | PCA | 7.57 | 30.3 | 46.1 | 75.3 | 98.5 |
|  |  |  | DP | 4.28 | 52.5 | 65.6 | 73.1 | 99.3 |
| 3 | Medicines and clinical reference | 148 | ORCHA | 35.0 | 55.4 | 62.0 | 75.3 | 86.5 |
|  |  |  | UX | 41.3 | 70.1 | 75.3 | 79.7 | 86.9 |
|  |  |  | PCA | 9.16 | 38.3 | 50.9 | 80.1 | 95.2 |
|  |  |  | DP | 7.19 | 58.8 | 66.8 | 73.8 | 89.7 |
| 4 | Neurological | 136 | ORCHA | 32.0 | 57.0 | 65.0 | 75.0 | 92.0 |
|  |  |  | UX | 44.0 | 71.3 | 76.1 | 79.6 | 85.7 |
|  |  |  | PCA | 13.0 | 39.2 | 61.2 | 79.3 | 98.4 |
|  |  |  | DP | 7.19 | 54.7 | 64.2 | 72.2 | 90.6 |
| 5 | Pregnancy | 82 | ORCHA | 27.0 | 52.0 | 66.0 | 74.8 | 83.0 |
|  |  |  | UX | 32.2 | 71.3 | 75.8 | 79.6 | 88.3 |
|  |  |  | PCA | 12.8 | 33.9 | 56.1 | 76.2 | 92.0 |
|  |  |  | DP | 27.7 | 61.4 | 69.2 | 74.0 | 88.5 |
| 6 | Diabetes | 81 | ORCHA | 26.0 | 53.0 | 63.5 | 79.0 | 96.0 |
|  |  |  | UX | 65.6 | 75.8 | 78.1 | 81.0 | 92.7 |
|  |  |  | PCA | 9.16 | 35.4 | 55.1 | 80.7 | 98.5 |
|  |  |  | DP | 5.13 | 58.6 | 68.1 | 74.5 | 94.9 |
| 7 | Respiratory | 77 | ORCHA | 40.0 | 66.0 | 74.0 | 79.0 | 92.0 |
|  |  |  | UX | 45.0 | 69.6 | 76.0 | 81.8 | 87.6 |
|  |  |  | PCA | 17.7 | 59.2 | 78.9 | 85.4 | 95.6 |
|  |  |  | DP | 41.3 | 60.8 | 71.7 | 77.2 | 95.2 |
| 8 | Children’s health | 71 | ORCHA | 41.0 | 62.0 | 68.0 | 74.0 | 84.5 |
|  |  |  | UX | 60.1 | 69.8 | 74.4 | 81.0 | 88.3 |
|  |  |  | PCA | 16.1 | 54.0 | 64.0 | 78.2 | 92.0 |
|  |  |  | DP | 37.8 | 58.5 | 65.0 | 72.0 | 95.6 |
| 9 | Women's health | 67 | ORCHA | 30.0 | 47.0 | 57.0 | 63.8 | 83.0 |
|  |  |  | UX | 32.2 | 69.1 | 72.2 | 77.4 | 86.9 |
|  |  |  | PCA | 14.5 | 25.6 | 36.4 | 61.3 | 92.3 |
|  |  |  | DP | 6.34 | 49.3 | 65.9 | 73.8 | 88.5 |
| 10 | Sexual health | 58 | ORCHA | 31.0 | 47.0 | 57.0 | 67.5 | 78.0 |
|  |  |  | UX | 32.2 | 68.9 | 72.5 | 78.6 | 85.4 |
|  |  |  | PCA | 14.8 | 25.2 | 35.9 | 60.4 | 85.7 |
|  |  |  | DP | 16.5 | 54.6 | 65.2 | 73.9 | 89.1 |
| 11 | Ophthalmology | 56 | ORCHA | 30.0 | 40.8 | 48.0 | 54.0 | 86.0 |
|  |  |  | UX | 37.9 | 65.6 | 69.7 | 73.1 | 81.8 |
|  |  |  | PCA | 9.16 | 16.1 | 28.8 | 40.2 | 89.3 |
|  |  |  | DP | 24.5 | 44.7 | 59.3 | 67.3 | 85.7 |
| 12 | Utilities or Administration | 55 | ORCHA | 39.0 | 56.3 | 64.5 | 80.0 | 85.0 |
|  |  |  | UX | 49.2 | 74.5 | 78.1 | 82.5 | 92.7 |
|  |  |  | PCA | 13.0 | 39.2 | 59.8 | 83.4 | 90.6 |
|  |  |  | DP | 7.19 | 56.1 | 70.5 | 78.4 | 85.4 |
| 13 | Cancer | 54 | ORCHA | 23.0 | 62.1 | 68.0 | 80.5 | 94.0 |
|  |  |  | UX | 50.1 | 71.4 | 76.9 | 81.6 | 92.7 |
|  |  |  | PCA | 7.57 | 51.8 | 68.4 | 84.8 | 97.4 |
|  |  |  | DP | 31.5 | 62.0 | 69.2 | 81.2 | 93.1 |
| 14 | Musculoskeletal disorders | 53 | ORCHA | 35.0 | 58.0 | 67.0 | 75.5 | 91.0 |
|  |  |  | UX | 38.8 | 73.0 | 76.1 | 79.6 | 87.3 |
|  |  |  | PCA | 13.0 | 38.6 | 58.9 | 79.4 | 96.1 |
|  |  |  | DP | 17.2 | 62.1 | 67.8 | 75.5 | 90.7 |
| 15 | Neurodiverse | 52 | ORCHA | 36.0 | 57.6 | 66.3 | 73.3 | 86.0 |
|  |  |  | UX | 55.7 | 71.9 | 75.6 | 79.4 | 84.7 |
|  |  |  | PCA | 16.1 | 43.2 | 68.2 | 80.0 | 95.3 |
|  |  |  | DP | 37.1 | 53.2 | 65.0 | 73.1 | 88.3 |
| 16 | Pain management | 44 | ORCHA | 26.0 | 49.8 | 62.8 | 76.0 | 84.0 |
|  |  |  | UX | 38.8 | 71.5 | 76.0 | 81.0 | 88.3 |
|  |  |  | PCA | 7.14 | 28.7 | 51.1 | 79.3 | 95.3 |
|  |  |  | DP | 17.2 | 55.2 | 63.7 | 75.0 | 89.9 |
| 17 | Cardiology | 34 | ORCHA | 47.0 | 58.3 | 68.5 | 79.4 | 92.0 |
|  |  |  | UX | 65.5 | 71.4 | 78.7 | 82.5 | 87.6 |
|  |  |  | PCA | 13.0 | 51.0 | 67.8 | 84.1 | 98.4 |
|  |  |  | DP | 44.2 | 59.9 | 68.5 | 75.9 | 90.6 |
| 18 | Dermatology | 29 | ORCHA | 23.0 | 46.0 | 63.0 | 66.0 | 85.0 |
|  |  |  | UX | 50.1 | 70.2 | 74.2 | 78.1 | 86.9 |
|  |  |  | PCA | 7.57 | 34.1 | 47.7 | 68.4 | 86.1 |
|  |  |  | DP | 4.62 | 46.1 | 62.8 | 70.2 | 82.8 |
| 19 | Dental | 25 | ORCHA | 32.0 | 53.0 | 59.0 | 75.0 | 84.5 |
|  |  |  | UX | 47.8 | 68.0 | 73.4 | 80.3 | 83.2 |
|  |  |  | PCA | 13.6 | 35.1 | 47.7 | 76.1 | 92.0 |
|  |  |  | DP | 30.8 | 43.0 | 64.2 | 67.2 | 77.5 |
| 20 | Gastrointestinal | 24 | ORCHA | 36.0 | 48.8 | 69.0 | 76.5 | 86.0 |
|  |  |  | UX | 42.0 | 69.0 | 72.9 | 79.9 | 86.9 |
|  |  |  | PCA | 12.2 | 26.4 | 66.9 | 79.7 | 94.8 |
|  |  |  | DP | 17.2 | 61.0 | 72.3 | 77.1 | 88.7 |
| 21 | Ear, Nose,  Throat, Mouth | 23 | ORCHA | 39.0 | 47.0 | 60.5 | 69.0 | 78.0 |
|  |  |  | UX | 50.7 | 67.6 | 72.8 | 77.2 | 80.3 |
|  |  |  | PCA | 14.5 | 22.8 | 41.8 | 80.4 | 86.3 |
|  |  |  | DP | 16.5 | 49.9 | 61.6 | 71.6 | 79.6 |
| 22 | Social support network | 17 | ORCHA | 34.0 | 51.0 | 67.0 | 74.0 | 81.0 |
|  |  |  | UX | 51.9 | 67.1 | 75.8 | 81.0 | 85.4 |
|  |  |  | PCA | 15.7 | 35.7 | 57.7 | 74.4 | 82.0 |
|  |  |  | DP | 21.3 | 58.7 | 65.2 | 72.4 | 80.3 |
| 23 | Urology | 15 | ORCHA | 49.0 | 67.0 | 74.0 | 79.0 | 81.0 |
|  |  |  | UX | 68.0 | 71.1 | 75.2 | 81.0 | 81.0 |
|  |  |  | PCA | 13.0 | 55.9 | 73.0 | 88.9 | 92.4 |
|  |  |  | DP | 53.2 | 63.2 | 69.3 | 73.5 | 86.9 |
| 24 | First aid | 14 | ORCHA | 48.0 | 60.8 | 70.5 | 76.0 | 84.0 |
|  |  |  | UX | 65.6 | 72.5 | 75.8 | 78.0 | 84.0 |
|  |  |  | PCA | 31.9 | 46.1 | 81.7 | 85.4 | 88.9 |
|  |  |  | DP | 44.9 | 56.7 | 68.1 | 73.5 | 80.3 |
| 25 | Allergy | 14 | ORCHA | 35.0 | 44.3 | 55.8 | 62.0 | 78.0 |
|  |  |  | UX | 69.6 | 73.3 | 76.3 | 78.1 | 84.0 |
|  |  |  | PCA | 14.8 | 30.9 | 45.4 | 55.1 | 82.5 |
|  |  |  | DP | 8.39 | 39.7 | 57.2 | 67.1 | 74.3 |
| 26 | Older adult | 13 | ORCHA | 18.0 | 55.0 | 61.0 | 75.0 | 77.5 |
|  |  |  | UX | 53.3 | 66.3 | 73.8 | 76.0 | 82.6 |
|  |  |  | PCA | 34.7 | 46.2 | 69.8 | 79.3 | 84.1 |
|  |  |  | DP | 41.3 | 58.6 | 59.3 | 65.0 | 73.7 |

Tier B

| **Index** | **Healthcare domain categories** | **Sample size (n)** | **Scores** | **0%** | **25%** | **50%**  **(median)** | **75%** | **100%** |
| --- | --- | --- | --- | --- | --- | --- | --- | --- |
| TB_1 | Healthy living | 436 | ORCHA | 30 | 49 | 61 | 72 | 89 |
|  |  |  | UX | 38.2 | 71.2 | 76.0 | 80.1 | 88.3 |
|  |  |  | PCA | 11.4 | 25.5 | 47.7 | 74.6 | 97.3 |
|  |  |  | DP | 15.1 | 54.2 | 65.0 | 72.8 | 88.3 |
| TB_2 | Mental health | 332 | ORCHA | 28.0 | 52.0 | 62.0 | 73.0 | 90.5 |
|  |  |  | UX | 32.6 | 70.2 | 75.2 | 79.6 | 88.3 |
|  |  |  | PCA | 11.4 | 30.3 | 47.7 | 75.3 | 95.3 |
|  |  |  | DP | 16.5 | 52.5 | 65.0 | 73.1 | 99.3 |
| TB_3 | Neurological | 104 | ORCHA | 38.0 | 60.4 | 68.0 | 75.0 | 84.0 |
|  |  |  | UX | 53.3 | 72.1 | 76.1 | 80.1 | 85.7 |
|  |  |  | PCA | 13.0 | 47.7 | 69.8 | 79.3 | 92.0 |
|  |  |  | DP | 7.19 | 54.7 | 61.9 | 70.7 | 88.3 |
| TB_4 | Medicines and clinical reference | 95 | ORCHA | 39.0 | 58.5 | 68.0 | 77.0 | 86.5 |
|  |  |  | UX | 49.2 | 70.2 | 77.3 | 80.9 | 84.7 |
|  |  |  | PCA | 16.1 | 50.9 | 65.0 | 84.9 | 95.2 |
|  |  |  | DP | 7.19 | 59.3 | 65.9 | 73.8 | 89.7 |
| TB_5 | Children’s health | 66 | ORCHA | 41.0 | 62.0 | 68.0 | 73.8 | 84.5 |
|  |  |  | UX | 60.1 | 70.0 | 74.5 | 81.6 | 88.3 |
|  |  |  | PCA | 16.1 | 54.0 | 63.5 | 77.6 | 92.0 |
|  |  |  | DP | 37.8 | 57.1 | 65.0 | 71.5 | 95.6 |
| TB_6 | Pregnancy | 56 | ORCHA | 27.0 | 62.8 | 70.8 | 76.0 | 83.0 |
|  |  |  | UX | 32.2 | 71.6 | 77.5 | 81.4 | 88.3 |
|  |  |  | PCA | 13.0 | 50.1 | 64.0 | 77.8 | 90.5 |
|  |  |  | DP | 31.5 | 58.6 | 67.8 | 71.8 | 86.8 |
| TB_7 | Respiratory | 49 | ORCHA | 50 | 68 | 73 | 78 | 89 |
|  |  |  | UX | 47.0 | 70.0 | 76.7 | 82.5 | 86.9 |
|  |  |  | PCA | 17.7 | 59.8 | 80.9 | 84.9 | 90.5 |
|  |  |  | DP | 41.3 | 60.8 | 72.1 | 77.2 | 95.2 |
| TB_8 | Neurodiverse | 48 | ORCHA | 48.0 | 59.5 | 67.5 | 74.3 | 86.0 |
|  |  |  | UX | 55.7 | 72.7 | 75.9 | 79.4 | 84.7 |
|  |  |  | PCA | 16.1 | 46.9 | 71.9 | 82.0 | 95.3 |
|  |  |  | DP | 37.9 | 54.0 | 65.0 | 74.0 | 88.3 |
| TB_9 | Utilities or Administration | 41 | ORCHA | 39 | 58 | 70 | 81 | 85 |
|  |  |  | UX | 49.2 | 73.1 | 78.1 | 82.5 | 92.7 |
|  |  |  | PCA | 13.0 | 42.0 | 69.4 | 84.1 | 90.6 |
|  |  |  | DP | 7.19 | 56.1 | 72.1 | 78.6 | 85.4 |
| TB_10 | Cancer | 37 | ORCHA | 53 | 63 | 75 | 83 | 94 |
|  |  |  | UX | 53.6 | 71.6 | 78.9 | 85.4 | 92.7 |
|  |  |  | PCA | 33.0 | 57.3 | 78.0 | 85.7 | 97.4 |
|  |  |  | DP | 45.2 | 62.0 | 69.8 | 81.5 | 93.1 |
| TB_11 | Women's health | 36 | ORCHA | 30.0 | 47.0 | 58.3 | 63.9 | 78.0 |
|  |  |  | UX | 32.2 | 67.8 | 71.7 | 78.1 | 85.4 |
|  |  |  | PCA | 14.5 | 32.7 | 48.5 | 63.4 | 85.7 |
|  |  |  | DP | 8.05 | 48.7 | 58.4 | 69.1 | 83.9 |
| TB_12 | Pain management | 35 | ORCHA | 34.0 | 49.5 | 63.5 | 76.0 | 83.0 |
|  |  |  | UX | 38.8 | 71.6 | 76.0 | 81.0 | 88.3 |
|  |  |  | PCA | 13.0 | 31.9 | 60.0 | 79.3 | 95.3 |
|  |  |  | DP | 17.2 | 54.9 | 63.0 | 73.6 | 82.4 |
| TB_13 | Diabetes | 33 | ORCHA | 43.0 | 56.0 | 67.5 | 77.0 | 84.0 |
|  |  |  | UX | 66.5 | 76.0 | 78.1 | 79.6 | 87.6 |
|  |  |  | PCA | 19.3 | 36.8 | 59.8 | 79.8 | 94.8 |
|  |  |  | DP | 39.4 | 47.6 | 64.1 | 72.1 | 84.6 |
| TB_14 | Musculoskeletal disorders | 30 | ORCHA | 35.0 | 61.9 | 67.0 | 74.8 | 84.0 |
|  |  |  | UX | 38.8 | 74.9 | 77.0 | 79.6 | 86.5 |
|  |  |  | PCA | 16.1 | 49.7 | 61.4 | 77.7 | 94.8 |
|  |  |  | DP | 17.2 | 57.8 | 65.4 | 70.2 | 81.3 |
| TB_15 | Ophthalmology | 30 | ORCHA | 38.0 | 43.0 | 50.0 | 55.5 | 70.0 |
|  |  |  | UX | 45.4 | 66.4 | 69.2 | 71.4 | 81.8 |
|  |  |  | PCA | 14.5 | 16.1 | 29.5 | 45.2 | 85.7 |
|  |  |  | DP | 24.5 | 45.9 | 57.4 | 64.9 | 77.0 |
| TB_16 | Sexual health | 27 | ORCHA | 32.0 | 54.5 | 62.0 | 69.5 | 78.0 |
|  |  |  | UX | 32.2 | 68.6 | 76.1 | 82.5 | 85.4 |
|  |  |  | PCA | 15.1 | 38.4 | 58.7 | 62.0 | 85.7 |
|  |  |  | DP | 16.5 | 59.3 | 63.3 | 66.7 | 89.1 |
| TB_17 | Dental | 20 | ORCHA | 38.0 | 54.4 | 59.5 | 75.0 | 84.5 |
|  |  |  | UX | 47.8 | 68.5 | 73.6 | 77.4 | 83.2 |
|  |  |  | PCA | 16.1 | 44.0 | 55.7 | 77.1 | 92.0 |
|  |  |  | DP | 30.8 | 43.3 | 64.8 | 67.5 | 77.5 |
| TB_18 | Ear, Nose, Throat, Mouth | 17 | ORCHA | 39 | 45 | 55 | 71 | 78 |
|  |  |  | UX | 50.7 | 66.4 | 70.0 | 76.7 | 80.3 |
|  |  |  | PCA | 14.5 | 19.3 | 41.8 | 82.0 | 86.3 |
|  |  |  | DP | 17.2 | 57.0 | 60.2 | 68.8 | 79.6 |
| TB_19 | Social support network | 16 | ORCHA | 36 | 51 | 68 | 74 | 81 |
|  |  |  | UX | 51.9 | 68.2 | 76.6 | 81.0 | 85.4 |
|  |  |  | PCA | 25.6 | 39.1 | 59.0 | 75.6 | 82.0 |
|  |  |  | DP | 21.3 | 58.7 | 65.2 | 73.1 | 80.3 |
| TB_20 | Dermatology | 16 | ORCHA | 45.0 | 63.4 | 64.3 | 66.5 | 85.0 |
|  |  |  | UX | 62.2 | 71.5 | 76.4 | 81.4 | 86.9 |
|  |  |  | PCA | 42.9 | 50.1 | 60.2 | 76.5 | 86.1 |
|  |  |  | DP | 4.62 | 45.2 | 63.3 | 73.5 | 82.8 |
| TB_21 | Cardiology | 16 | ORCHA | 47.0 | 56.5 | 66.0 | 80.1 | 83.0 |
|  |  |  | UX | 66.5 | 73.1 | 81.0 | 82.5 | 87.6 |
|  |  |  | PCA | 13.0 | 47.7 | 64.0 | 84.1 | 87.3 |
|  |  |  | DP | 44.2 | 56.8 | 62.9 | 70.7 | 77.2 |
| TB_22 | First aid | 14 | ORCHA | 48.0 | 60.8 | 70.5 | 76.0 | 84.0 |
|  |  |  | UX | 65.6 | 72.5 | 75.8 | 78.0 | 84.0 |
|  |  |  | PCA | 31.9 | 46.1 | 81.7 | 85.4 | 88.9 |
|  |  |  | DP | 44.9 | 56.7 | 68.1 | 73.5 | 80.3 |
| TB_23 | Gastrointestinal | 13 | ORCHA | 36 | 48 | 56 | 74 | 84 |
|  |  |  | UX | 42.0 | 69.6 | 73.0 | 77.5 | 86.9 |
|  |  |  | PCA | 12.2 | 19.3 | 42.9 | 75.3 | 94.8 |
|  |  |  | DP | 17.2 | 58.7 | 67.7 | 72.6 | 74.5 |
| TB_24 | Urology | 12 | ORCHA | 49.0 | 64.5 | 71.5 | 74.4 | 81.0 |
|  |  |  | UX | 68.0 | 74.1 | 80.6 | 81.0 | 81.0 |
|  |  |  | PCA | 13.0 | 54.3 | 73.0 | 78.5 | 88.9 |
|  |  |  | DP | 53.2 | 66.4 | 69.6 | 77.0 | 86.9 |
| TB_25 | Older adult | 10 | ORCHA | 18.0 | 57.3 | 66.5 | 74.3 | 77.0 |
|  |  |  | UX | 53.3 | 73.1 | 74.2 | 76.0 | 81.7 |
|  |  |  | PCA | 34.7 | 45.0 | 73.8 | 79.3 | 82.5 |
|  |  |  | DP | 41.3 | 57.5 | 61.5 | 67.4 | 73.7 |
| TB_26 | Allergy | 9 | ORCHA | 43 | 48 | 58 | 63 | 78 |
|  |  |  | UX | 71.2 | 75.8 | 76.7 | 78.1 | 84.0 |
|  |  |  | PCA | 16.1 | 47.7 | 55.1 | 55.6 | 82.5 |
|  |  |  | DP | 8.39 | 37.1 | 61.3 | 65.4 | 69.3 |

Tier C

| **Index** | **Categories** | **Sample size (n)** | **Scores** | **0%** | **25%** | **50%**  **(median)** | **75%** | **100%** |
| --- | --- | --- | --- | --- | --- | --- | --- | --- |
| TC_1 | Healthy living | 106 | ORCHA | 31 | 51 | 60 | 74 | 96 |
|  |  |  | UX | 59.9 | 69.6 | 73.3 | 79.6 | 94.2 |
|  |  |  | PCA | 14.8 | 34.4 | 46.6 | 74.9 | 98.5 |
|  |  |  | DP | 6.34 | 58.3 | 67.5 | 75.4 | 94.9 |
| TC_2 | Mental health | 102 | ORCHA | 25.0 | 46.3 | 55.0 | 71.0 | 96.0 |
|  |  |  | UX | 27.4 | 68.6 | 72.5 | 77.8 | 92.7 |
|  |  |  | PCA | 7.57 | 31.1 | 39.5 | 68.0 | 98.5 |
|  |  |  | DP | 4.28 | 51.7 | 67.3 | 75.3 | 94.9 |
| TC_3 | Medicines and clinical reference | 49 | ORCHA | 35.0 | 46.0 | 55.5 | 60.0 | 85.0 |
|  |  |  | UX | 41.3 | 69.6 | 72.8 | 78.1 | 86.9 |
|  |  |  | PCA | 9.16 | 29.1 | 38.3 | 46.5 | 93.6 |
|  |  |  | DP | 30.6 | 57.0 | 66.8 | 70.2 | 83.2 |
| TC_4 | Diabetes | 48 | ORCHA | 26 | 50 | 63 | 79 | 96 |
|  |  |  | UX | 65.6 | 74.2 | 78.1 | 82.0 | 92.7 |
|  |  |  | PCA | 9.16 | 34.1 | 47.8 | 82.8 | 98.5 |
|  |  |  | DP | 5.13 | 62.0 | 68.2 | 74.5 | 94.9 |
| TC_5 | Women's health | 31 | ORCHA | 31.0 | 47.0 | 57.0 | 63.5 | 83.0 |
|  |  |  | UX | 55.0 | 70.0 | 72.8 | 75.8 | 86.9 |
|  |  |  | PCA | 14.8 | 25.2 | 35.9 | 46.5 | 92.3 |
|  |  |  | DP | 6.34 | 60.2 | 73.3 | 75.8 | 88.5 |
| TC_6 | Sexual health | 31 | ORCHA | 31.0 | 46.5 | 51.0 | 57.0 | 77.0 |
|  |  |  | UX | 64.0 | 69.8 | 71.5 | 75.8 | 85.4 |
|  |  |  | PCA | 14.8 | 23.6 | 27.5 | 36.1 | 75.5 |
|  |  |  | DP | 27.7 | 53.9 | 70.5 | 77.3 | 88.5 |
| TC_7 | Neurological | 30 | ORCHA | 32.0 | 54.3 | 57.0 | 63.0 | 92.0 |
|  |  |  | UX | 44.0 | 68.9 | 74.9 | 78.1 | 85.4 |
|  |  |  | PCA | 16.6 | 32.1 | 39.4 | 65.9 | 98.4 |
|  |  |  | DP | 15.1 | 51.7 | 66.8 | 76.3 | 90.6 |
| TC_8 | Respiratory | 28 | ORCHA | 40.0 | 63.0 | 74.5 | 79.3 | 92.0 |
|  |  |  | UX | 45.0 | 69.6 | 73.6 | 81.2 | 87.6 |
|  |  |  | PCA | 18.7 | 51.2 | 78.5 | 86.8 | 95.6 |
|  |  |  | DP | 50.2 | 61.0 | 68.6 | 76.8 | 92.8 |
| TC_9 | Pregnancy | 26 | ORCHA | 31.0 | 48.3 | 55.0 | 58.8 | 79.0 |
|  |  |  | UX | 61.6 | 70.0 | 72.8 | 75.8 | 85.4 |
|  |  |  | PCA | 12.8 | 23.9 | 33.7 | 39.7 | 92.0 |
|  |  |  | DP | 27.7 | 63.5 | 73.3 | 74.6 | 88.5 |
| TC_10 | Ophthalmology | 26 | ORCHA | 30.0 | 37.5 | 43.3 | 51.8 | 86.0 |
|  |  |  | UX | 37.9 | 64.8 | 69.8 | 74.1 | 80.1 |
|  |  |  | PCA | 9.16 | 14.4 | 28.8 | 35.6 | 89.3 |
|  |  |  | DP | 25.5 | 41.9 | 60.6 | 71.2 | 85.7 |
| TC_11 | Musculoskeletal disorders | 23 | ORCHA | 44.0 | 57.0 | 68.0 | 84.5 | 91.0 |
|  |  |  | UX | 56.5 | 71.3 | 74.2 | 79.5 | 87.3 |
|  |  |  | PCA | 13.0 | 34.7 | 52.6 | 91.2 | 96.1 |
|  |  |  | DP | 59.3 | 65.4 | 75.5 | 84.3 | 90.7 |
| TC_12 | Cardiology | 18 | ORCHA | 55.0 | 59.0 | 72.5 | 79.0 | 92.0 |
|  |  |  | UX | 65.5 | 71.3 | 74.2 | 81.4 | 86.9 |
|  |  |  | PCA | 37.8 | 52.7 | 73.4 | 91.8 | 98.4 |
|  |  |  | DP | 55.3 | 64.9 | 71.0 | 77.4 | 90.6 |
| TC_13 | Cancer | 17 | ORCHA | 23 | 46 | 64 | 70 | 83 |
|  |  |  | UX | 50.1 | 66.4 | 72.9 | 78.6 | 85.4 |
|  |  |  | PCA | 7.57 | 30.4 | 51.8 | 68.4 | 92.3 |
|  |  |  | DP | 31.5 | 59.6 | 68.5 | 74.2 | 85.8 |
| TC_14 | Utilities or Administration | 14 | ORCHA | 40.0 | 52.3 | 57.0 | 61.8 | 82.0 |
|  |  |  | UX | 71.3 | 76.7 | 80.3 | 83.4 | 86.9 |
|  |  |  | PCA | 22.9 | 29.9 | 41.6 | 52.6 | 84.5 |
|  |  |  | DP | 47.2 | 53.1 | 67.9 | 70.7 | 80.5 |
| TC_15 | Dermatology | 13 | ORCHA | 23 | 39 | 46 | 55 | 68 |
|  |  |  | UX | 50.1 | 66.4 | 72.9 | 74.5 | 79.6 |
|  |  |  | PCA | 7.57 | 18.6 | 25.0 | 34.2 | 68.4 |
|  |  |  | DP | 31.5 | 48.8 | 59.6 | 68.5 | 75.2 |
| TC_16 | Gastrointestinal | 11 | ORCHA | 40 | 63 | 71 | 78 | 86 |
|  |  |  | UX | 56.5 | 68.4 | 72.9 | 81.0 | 86.9 |
|  |  |  | PCA | 12.6 | 47.9 | 73.0 | 81.0 | 84.5 |
|  |  |  | DP | 54.1 | 70.2 | 79.6 | 85.8 | 88.7 |
| TC_17 | Pain management | 9 | ORCHA | 26 | 50 | 59 | 74 | 84 |
|  |  |  | UX | 40.9 | 56.5 | 75.8 | 80.3 | 84.0 |
|  |  |  | PCA | 7.14 | 25.6 | 43.4 | 78.1 | 92.7 |
|  |  |  | DP | 46.7 | 62.0 | 67.1 | 76.2 | 89.9 |
| TC_18 | Ear, Nose, Throat, Mouth | 6 | ORCHA | 39.0 | 52.6 | 61.8 | 66.0 | 75.5 |
|  |  |  | UX | 69.6 | 71.1 | 74.0 | 77.7 | 79.6 |
|  |  |  | PCA | 25.9 | 31.1 | 55.8 | 76.6 | 84.8 |
|  |  |  | DP | 16.5 | 44.9 | 65.6 | 72.6 | 77.4 |
| TC_19 | Dental | 5 | ORCHA | 32.0 | 32.0 | 44.0 | 56.0 | 78.5 |
|  |  |  | UX | 61.5 | 63.1 | 68.4 | 80.3 | 81.0 |
|  |  |  | PCA | 13.6 | 13.6 | 28.6 | 40.9 | 81.9 |
|  |  |  | DP | 42.4 | 42.4 | 52.6 | 64.2 | 71.7 |
| TC_20 | Children’s health | 5 | ORCHA | 56 | 70 | 70 | 76 | 77 |
|  |  |  | UX | 64.8 | 66.4 | 69.8 | 71.3 | 81.0 |
|  |  |  | PCA | 40.9 | 67.4 | 67.4 | 90.9 | 90.9 |
|  |  |  | DP | 59.3 | 59.3 | 65.9 | 73.3 | 73.3 |
| TC_21 | Allergy | 5 | ORCHA | 35 | 42 | 42 | 61 | 62 |
|  |  |  | UX | 69.6 | 69.6 | 72.9 | 82.5 | 84.0 |
|  |  |  | PCA | 14.8 | 27.5 | 27.5 | 41.1 | 41.1 |
|  |  |  | DP | 33.9 | 47.2 | 48.1 | 74.3 | 74.3 |
| TC_22 | Urology | 3 | ORCHA | 79 | 79 | 79 | 79 | 79 |
|  |  |  | UX | 68.0 | 68.4 | 68.8 | 70.4 | 72.0 |
|  |  |  | PCA | 90.8 | 91.4 | 92.0 | 92.2 | 92.4 |
|  |  |  | DP | 61.6 | 63.2 | 64.7 | 65.2 | 65.7 |
| TC_23 | Neurodiverse | 3 | ORCHA | 36 | 41 | 46 | 50 | 54 |
|  |  |  | UX | 65.5 | 66.3 | 67.2 | 74.0 | 80.9 |
|  |  |  | PCA | 23.4 | 28.4 | 33.5 | 36.0 | 38.5 |
|  |  |  | DP | 37.1 | 41.0 | 44.8 | 58.4 | 71.9 |
| TC_24 | Older adult | 2 | ORCHA | 55 | 55 | 55 | 55 | 55 |
|  |  |  | UX | 64.7 | 65.1 | 65.5 | 65.9 | 66.3 |
|  |  |  | PCA | 48.1 | 48.1 | 48.1 | 48.1 | 48.1 |
|  |  |  | DP | 59.3 | 59.3 | 59.3 | 59.3 | 59.3 |
| TC_25 | Social support network | 1 | ORCHA | 34 | 34 | 34 | 34 | 34 |
|  |  |  | UX | 62.5 | 62.5 | 62.5 | 62.5 | 62.5 |
|  |  |  | PCA | 15.7 | 15.7 | 15.7 | 15.7 | 15.7 |
|  |  |  | DP | 46.7 | 46.7 | 46.7 | 46.7 | 46.7 |
