# Supplementary material for: Quality of Digital Health Interventions Across Different Health Care Domains: Secondary Data Analysis Study
Source: JMIR Mhealth Uhealth. 2023 Nov 23;11:e47043. doi: 10.2196/47043 (PMC10704310; doi:10.2196/47043)
Supplement: Multimedia Appendix 5 [file mhealth_v11i1e47043_app5.docx]

## Appendix 5 – Rank of DHIs

**Appendix 7 Table 1**: DHI in descending order based on ORCHA median score.

| **Rank** | **All DHI** | | **Tier B DHI** | | **Tier C DHI** | |
| --- | --- | --- | --- | --- | --- | --- |
| 1 | Respiratory | m=74.0, n=77 | Cancer | m=75.0, n=37 | Urology | m=79.0, n =3 |
| 2 | Urology | m=74.0, n=15 | Respiratory | m=73.0, n=49 | Respiratory | m=74.5, n=28 |
| 3 | First aid | m=70.5, n=14 | Urology | m=71.5, n=12 | Cardiology | m=72.5, n=18 |
| 4 | Gastrointestinal | m=69.0, n=24 | Pregnancy | m=70.8, n=56 | Gastrointestinal | m=71.0, n =11 |
| 5 | Cardiology | m=68.5, n=34 | First Aid | m=70.5, n=14 | Children’s health | m=70.0, n=5 |
| 6 | Cancer | m=68.0, n=54 | UA | m=70.0, n=41 | MD | m=68.0, n=23 |
| 7 | Children’s health | m=68.0, n=71 | Children’s health | m=68.0, n=66 | Cancer | m=64.0, n=17 |
| 8 | MD | m=67.0, n=53 | SSN | m=68.0, n=16 | Diabetes | m=63.0, n=48 |
| 9 | SSN | m=67.0, n=17 | Neurological | m=68.0, n=104 | ENTM | m=61.8, n=6 |
| 10 | Neurodiverse | m=66.3, n=52 | MaCR | m=68.0, n=95 | Healthy living | m=60.0, n=106 |
| 11 | Pregnancy | m=66.0, n=82 | Neurodiverse | m=67.5, n=48 | PM | m=59.0, n=9 |
| 12 | Neurological | m=65.0, n=136 | Diabetes | m=67.5, n=33 | Women’s health | m=57.0, n=31 |
| 13 | UA | m=64.5, n=55 | MD | m=67.0, n=30 | Neurological | m=57.0, n=30 |
| 14 | Diabetes | m=63.5, n=81 | OA | m=66.5, n=10 | UA | m=57.0, n=14 |
| 15 | Dermatology | m=63.0, n=29 | Cardiology | m=66.0, n=16 | MaCR | m=55.5, n=49 |
| 16 | PM | m=62.8, n=44 | Dermatology | m=64.3, n=16 | Mental health | m=55.0, n =102 |
| 17 | MaCR | m=62.0, n=148 | PM | m=63.5, n=35 | Pregnancy | m=55.0, n=26 |
| 18 | OA | m=61.0, n=13 | Mental health | m=62.0, n=332 | OA | m=55.0, n=2 |
| 19 | Healthy living | m=61.0, n=548 | Sexual health | m=62.0, n=27 | Sexual health | m=51.0, n=31 |
| 20 | ENTM | m=60.5, n=23 | Healthy living | m=61.0, n=436 | Dermatology | m=46.0, n=13 |
| 21 | Mental health | m=60.0, n=436 | Dental | m=59.5, n=20 | Neurodiverse | m=46.0, n=3 |
| 22 | Dental | m=59.0, n=25 | Women’s health | m=58.3, n=36 | Dental | m=44.0, n=5 |
| 23 | Sexual health | m=57.0, n=58 | Allergy | m=58.0, n=9 | Ophthalmology | m=43.3, n=26 |
| 24 | Women’s health | m=57.0, n=67 | Gastrointestinal | m=56.0, n=13 | Allergy | m=42.0, n=5 |
| 25 | Allergy | m=55.8, n=14 | ENTM | m=55.0, n=17 | SSN | m=34.0, n=1 |
| 26 | Ophthalmology | m=48.0, n=56 | Ophthalmology | m=50.0, n=30 | First aid* | NA |

*No First aid category in Tier C

**Appendix 7 Table 2**: DHI in descending order based on UX median score.

| **Rank** | **All DHI** | | **Tier B DHI** | | **Tier C DHI** | |
| --- | --- | --- | --- | --- | --- | --- |
| 1 | Cardiology | m=78.7, n=34 | Cardiology | m=81.0, n=16 | UA | m=80.3, n=14 |
| 2 | Diabetes | m=78.1, n=81 | Urology | m=80.6, n=12 | Diabetes | m=78.1, n=48 |
| 3 | UA | m=78.1, n=55 | Cancer | m=78.9, n=37 | PM | m=75.8, n=9 |
| 4 | Cancer | m=76.9, n=54 | Diabetes | m=78.1, n=33 | Neurological | m=74.9, n=30 |
| 5 | Allergy | m=76.3, n=14 | UA | m=78.1, n=41 | MD | m=74.2, n=23 |
| 6 | MD | m=76.1, n=53 | Pregnancy | m=77.5, n=56 | Cardiology | m=74.2, n=18 |
| 7 | Neurological | m=76.1, n=136 | MaCR | m=77.3, n=95 | ENTM | m=74.0, n=6 |
| 8 | Respiratory | m=76.0, n=77 | MD | m=77.0, n=30 | Respiratory | m=73.6, n=28 |
| 9 | PM | m=76.0, n=44 | Allergy | m=76.7, n=9 | Healthy living | m=73.3, n=106 |
| 10 | First aid | m=75.8, n=14 | Respiratory | m=76.7, n=49 | Allergy | m=72.9, n=5 |
| 11 | Pregnancy | m=75.8, n=82 | SSN | m=76.6, n=16 | Cancer | m=72.9, n=17 |
| 12 | SSN | m=75.8, n=17 | Dermatology | m=76.4, n=16 | Gastrointestinal | m=72.9, n=11 |
| 13 | Neurodiverse | m=75.6, n=52 | Neurological | m=76.1, n=104 | Dermatology | m=72.9, n=13 |
| 14 | MaCR | m=75.3, n=148 | Sexual health | m=76.1, n=27 | Pregnancy | m=72.8, n=26 |
| 15 | Healthy living | m=75.2, n=548 | Healthy living | m=76.0, n=436 | MaCR | m=72.8, n=49 |
| 16 | Mental health | m=75.2, n=436 | PM | m=76.0, n=35 | Women’s health | m=72.8, n=31 |
| 17 | Urology | m=75.2, n=15 | Neurodiverse | m=75.9, n=48 | Mental health | m=72.5, n=102 |
| 18 | Children’s health | m=74.4, n=71 | First aid | m=75.8, n=14 | Sexual health | m=71.5, n=31 |
| 19 | Dermatology | m=74.2, n=29 | Mental health | m=75.2, n=332 | Children’s health | m=69.8, n=5 |
| 20 | OA | m=73.8, n=13 | Children’s health | m=74.5, n=66 | Ophthalmology | m=69.8, n=26 |
| 21 | Dental | m=73.4, n=25 | OA | m=74.2, n=10 | Urology | m=68.8, n=3 |
| 22 | Gastrointestinal | m=72.9, n=24 | Dental | m=73.6, n=20 | Dental | m=68.4, n=5 |
| 23 | ENTM | m=72.8, n=23 | Gastrointestinal | m=73.0, n=13 | Neurodiverse | m=67.2, n=3 |
| 24 | Sexual health | m=72.5, n=58 | Women’s health | m=71.7, n=36 | OA | m=65.5, n=2 |
| 25 | Women’s health | m=72.2, n=67 | ENTM | m=70.0, n=17 | SSN | m=62.5, n=1 |
| 26 | Ophthalmology | m=69.7, n=56 | Ophthalmology | m=69.2, n=30 | First aid* | NA |

*No First Aid category in Tier C

**Appendix 7 Table 3**: DHI in descending order based on PCA median score.

| **Rank** | **All DHI** | | **Tier B DHI** | | **Tier C DHI** | |
| --- | --- | --- | --- | --- | --- | --- |
| 1 | First aid | m=81.7, n=14 | First aid | m=81.7, n=14 | Urology | m=92.0, n=3 |
| 2 | Respiratory | m=78.9, n=77 | Respiratory | m=80.9, n=49 | Respiratory | m=78.5, n=28 |
| 3 | Urology | m=73.0, n=15 | Cancer | m=78.0, n=37 | Cardiology | m=73.4, n=18 |
| 4 | OA | m=69.8, n=13 | OA | m=73.8, n=10 | Gastrointestinal | m=73.0, n=11 |
| 5 | Cancer | m=68.4, n=54 | Urology | m=73.0, n=12 | Children’s health | m=67.4, n=5 |
| 6 | Neurodiverse | m=68.2, n=52 | Neurodiverse | m=71.9, n=48 | ENTM | m=55.8, n=6 |
| 7 | Cardiology | m=67.8, n=34 | Neurological | m=69.8, n=104 | MD | m=52.6, n=23 |
| 8 | Gastrointestinal | m=66.9, n=24 | UA | m=69.4, n=41 | Cancer | m=51.8, n=17 |
| 9 | Children’s health | m=64.0, n=71 | MaCR | m=65.0, n=95 | OA | m=48.1, n=2 |
| 10 | Neurological | m=61.2, n=136 | Cardiology | m=64.0, n=16 | Diabetes | m=47.8, n=48 |
| 11 | UA | m=59.8, n=55 | Pregnancy | m=64.0, n=56 | Healthy living | m=46.6, n=106 |
| 12 | MD | m=58.9, n=53 | Children’s health | m=63.5, n=66 | PM | m=43.4, n=9 |
| 13 | SSN | m=57.7, n=17 | MD | m=61.4, n=30 | UA | m=41.6, n=14 |
| 14 | Pregnancy | m=56.1, n=82 | Dermatology | m=60.2, n=16 | Mental health | m=39.5, n=102 |
| 15 | Diabetes | m=55.1, n=81 | PM | m=60.0, n=35 | Neurological | m=39.4, n=30 |
| 16 | PM | m=51.1, n=44 | Diabetes | m=59.8, n=33 | MaCR | m=38.3, n=49 |
| 17 | MaCR | m=50.9, n=148 | SSN | m=59.0, n=16 | Women’s health | m=35.9, n=31 |
| 18 | Dental | m=47.7, n=25 | Sexual health | m=58.7, n=27 | Pregnancy | m=33.7, n=26 |
| 19 | Healthy living | m=47.7, n=548 | Dental | m=55.7, n=20 | Neurodiverse | m=33.5, n=3 |
| 20 | Dermatology | m=47.7, n=29 | Allergy | m=55.1, n=9 | Ophthalmology | m=28.8, n=26 |
| 21 | Mental health | m=46.1, n=436 | Women’s health | m=48.5, n=36 | Dental | m=28.6, n=5 |
| 22 | Allergy | m=45.4, n=14 | Mental health | m=47.7, n=332 | Allergy | m=27.5, n=5 |
| 23 | ENTM | m=41.8, n=23 | Healthy living | m=47.7, n=436 | Sexual health | m=27.5, n=31 |
| 24 | Women’s health | m=36.4, n=67 | Gastrointestinal | m=42.9, n=13 | Dermatology | m=25.0, n=13 |
| 25 | Sexual health | m=35.9, n=58 | ENTM | m=41.8, n=17 | SSN | m=15.7, n=1 |
| 26 | Ophthalmology | m=28.8, n=56 | Ophthalmology | m=29.5, n=30 | First aid* | NA |

*No First Aid category in Tier C

**Appendix 7 Table 4**: DHI in descending order based on DP median score.

| **Rank** | **All DHI** | | **Tier B DHI** | | **Tier C DHI** | |
| --- | --- | --- | --- | --- | --- | --- |
| 1 | Gastrointestinal | m=72.3, n=24 | Respiratory | m=72.1, n=49 | Gastrointestinal | m=79.6, n=11 |
| 2 | Respiratory | m=71.7, n=77 | UA | m=72.1, n=41 | MD | m=75.5, n=23 |
| 3 | UA | m=70.5, n=55 | Cancer | m=69.8, n=37 | Pregnancy | m=73.3, n=26 |
| 4 | Urology | m=69.3, n=15 | Urology | m=69.6, n=12 | Women’s health | m=73.3, n=31 |
| 5 | Cancer | m=69.2, n=54 | First aid | m=68.1, n=14 | Cardiology | m=71.0, n=18 |
| 6 | Pregnancy | m=69.2, n=82 | Pregnancy | m=67.8, n=56 | Sexual health | m=70.5, n=31 |
| 7 | Cardiology | m=68.5, n=34 | Gastrointestinal | m=67.7, n=13 | Respiratory | m=68.6, n=28 |
| 8 | Diabetes | m=68.1, n=81 | MaCR | m=65.9, n=95 | Cancer | m=68.5, n=17 |
| 9 | First aid | m=68.1, n=14 | MD | m=65.4, n=30 | Diabetes | m=68.2, n=48 |
| 10 | MD | m=67.8, n=53 | SSN | m=65.2, n=16 | UA | m=67.9, n=14 |
| 11 | MaCR | m=66.8, n=148 | Mental health | m=65.0, n=332 | Healthy living | m=67.5, n=106 |
| 12 | Women’s health | m=65.9, n=67 | Children’s health | m=65.0, n=66 | Mental health | m=67.3, n=102 |
| 13 | Mental health | m=65.6, n=436 | Healthy living | m=65.0, n=436 | PM | m=67.1, n=9 |
| 14 | Sexual health | m=65.2, n=58 | Neurodiverse | m=65.0, n=48 | Neurological | m=66.8, n=30 |
| 15 | SSN | m=65.2, n=17 | Dental | m=64.8, n=20 | MaCR | m=66.8, n=49 |
| 16 | Healthy living | m=65.0, n=548 | Diabetes | m=64.1, n=33 | Children’s health | m=65.9, n=5 |
| 17 | Children’s health | m=65.0, n=71 | Sexual health | m=63.3, n=27 | ENTM | m=65.6, n=6 |
| 18 | Neurodiverse | m=65.0, n=52 | Dermatology | m=63.3, n=16 | Urology | m=64.7, n=3 |
| 19 | Dental | m=64.2, n=25 | PM | m=63.0, n=35 | Ophthalmology | m=60.6, n=26 |
| 20 | Neurological | m=64.2, n=136 | Cardiology | m=62.9, n=16 | Dermatology | m=59.6, n=13 |
| 21 | PM | m=63.7, n=44 | Neurological | m=61.9, n=104 | OA | m=59.3, n=2 |
| 22 | Dermatology | m=62.8, n=29 | OA | m=61.5, n=10 | Dental | m=52.6, n=5 |
| 23 | ENTM | m=61.6, n=23 | Allergy | m=61.3, n=9 | Allergy | m=48.1, n=5 |
| 24 | OA | m=59.3, n=13 | ENTM | m=60.2, n=17 | SSN | m=46.7, n=1 |
| 25 | Ophthalmology | m=59.3, n=56 | Women’s health | m=58.4, n=36 | Neurodiverse | m=44.8, n=3 |
| 26 | Allergy | m=57.2, n=14 | Ophthalmology | m=57.4, n=30 | First aid* | NA |

*No First Aid category in Tier C
