# Supplementary material for: Quality of Digital Health Interventions Across Different Health Care Domains: Secondary Data Analysis Study
Source: JMIR Mhealth Uhealth. 2023 Nov 23;11:e47043. doi: 10.2196/47043 (PMC10704310; doi:10.2196/47043)
Supplement: Multimedia Appendix 6 [file mhealth_v11i1e47043_app6.docx]

Appendix 6 – Tables’ x and y axis are correspondent to index in appendix 2. Post hoc analysis with Dunn test (*P*-values adjusted using Holm’s method) for scores across healthcare domains, *P*<.05 and NAs are in red font.

**Appendix 3 table 1**: ORCHA score pairwise comparison.

|  | **1** | **2** | **3** | **4** | **5** | **6** | **7** | **8** | **9** | **10** | **11** | **12** | **13** | **14** | **15** | **16** | **17** | **18** | **19** | **20** | **21** | **22** | **23** | **24** | **25** | **26** |
| --- | --- | --- | --- | --- | --- | --- | --- | --- | --- | --- | --- | --- | --- | --- | --- | --- | --- | --- | --- | --- | --- | --- | --- | --- | --- | --- |
| **1** | NA |  |  |  |  |  |  |  |  |  |  |  |  |  |  |  |  |  |  |  |  |  |  |  |  |  |
| **2** | 1 | NA |  |  |  |  |  |  |  |  |  |  |  |  |  |  |  |  |  |  |  |  |  |  |  |  |
| **3** | 1 | 1 | NA |  |  |  |  |  |  |  |  |  |  |  |  |  |  |  |  |  |  |  |  |  |  |  |
| **4** | 1 | 1 | 1 | NA |  |  |  |  |  |  |  |  |  |  |  |  |  |  |  |  |  |  |  |  |  |  |
| **5** | 1 | 1 | 1 | 1 | NA |  |  |  |  |  |  |  |  |  |  |  |  |  |  |  |  |  |  |  |  |  |
| **6** | 1 | 1 | 1 | 1 | 1 | NA |  |  |  |  |  |  |  |  |  |  |  |  |  |  |  |  |  |  |  |  |
| **7** | <.001 | <.001 | .002 | .042 | .046 | .058 | NA |  |  |  |  |  |  |  |  |  |  |  |  |  |  |  |  |  |  |  |
| **8** | .074 | .094 | 1 | 1 | 1 | 1 | 1 | NA |  |  |  |  |  |  |  |  |  |  |  |  |  |  |  |  |  |  |
| **9** | 1 | 1 | .246 | .035 | .448 | .397 | <.001 | .001 | NA |  |  |  |  |  |  |  |  |  |  |  |  |  |  |  |  |  |
| **10** | 1 | 1 | .268 | .042 | .448 | .397 | <.001 | .001 | 1 | NA |  |  |  |  |  |  |  |  |  |  |  |  |  |  |  |  |
| **11** | <.001 | <.001 | <.001 | <.001 | <.001 | <.001 | <.001 | <.001 | .817 | 1 | NA |  |  |  |  |  |  |  |  |  |  |  |  |  |  |  |
| **12** | 1 | 1 | 1 | 1 | 1 | 1 | 1 | 1 | .097 | .098 | <.001 | NA |  |  |  |  |  |  |  |  |  |  |  |  |  |  |
| **13** | .020 | .025 | 1 | 1 | 1 | 1 | 1 | 1 | <.001 | <.001 | <.001 | 1 | NA |  |  |  |  |  |  |  |  |  |  |  |  |  |
| **14** | 1 | 1 | 1 | 1 | 1 | 1 | 1 | 1 | .030 | .031 | <.001 | 1 | 1 | NA |  |  |  |  |  |  |  |  |  |  |  |  |
| **15** | 1 | 1 | 1 | 1 | 1 | 1 | 1 | 1 | .131 | .131 | <.001 | 1 | 1 | 1 | NA |  |  |  |  |  |  |  |  |  |  |  |
| **16** | 1 | 1 | 1 | 1 | 1 | 1 | .097 | 1 | 1 | 1 | <.001 | 1 | 1 | 1 | 1 | NA |  |  |  |  |  |  |  |  |  |  |
| **17** | .176 | .198 | 1 | 1 | 1 | 1 | 1 | 1 | .003 | .003 | <.001 | 1 | 1 | 1 | 1 | 1 | NA |  |  |  |  |  |  |  |  |  |
| **18** | 1 | 1 | 1 | 1 | 1 | 1 | .002 | .726 | 1 | 1 | .944 | 1 | .239 | 1 | 1 | 1 | .406 | NA |  |  |  |  |  |  |  |  |
| **19** | 1 | 1 | 1 | 1 | 1 | 1 | .032 | 1 | 1 | 1 | .423 | 1 | 1 | 1 | 1 | 1 | 1 | 1 | NA |  |  |  |  |  |  |  |
| **20** | 1 | 1 | 1 | 1 | 1 | 1 | 1 | 1 | 1 | 1 | .004 | 1 | 1 | 1 | 1 | 1 | 1 | 1 | 1 | NA |  |  |  |  |  |  |
| **21** | 1 | 1 | 1 | 1 | 1 | 1 | .007 | 1 | 1 | 1 | 1 | 1 | .437 | 1 | 1 | 1 | .629 | 1 | 1 | 1 | NA |  |  |  |  |  |
| **22** | 1 | 1 | 1 | 1 | 1 | 1 | 1 | 1 | 1 | 1 | .144 | 1 | 1 | 1 | 1 | 1 | 1 | 1 | 1 | 1 | 1 | NA |  |  |  |  |
| **23** | 1 | 1 | 1 | 1 | 1 | 1 | 1 | 1 | .077 | .071 | <.001 | 1 | 1 | 1 | 1 | 1 | 1 | 1 | 1 | 1 | 1 | 1 | NA |  |  |  |
| **24** | 1 | 1 | 1 | 1 | 1 | 1 | 1 | 1 | .874 | .789 | .001 | 1 | 1 | 1 | 1 | 1 | 1 | 1 | 1 | 1 | 1 | 1 | 1 | NA |  |  |
| **25** | 1 | 1 | 1 | 1 | 1 | 1 | .008 | .601 | 1 | 1 | 1 | 1 | .238 | 1 | 1 | 1 | .304 | 1 | 1 | 1 | 1 | 1 | .550 | 1 | NA |  |
| **26** | 1 | 1 | 1 | 1 | 1 | 1 | 1 | 1 | 1 | 1 | .279 | 1 | 1 | 1 | 1 | 1 | 1 | 1 | 1 | 1 | 1 | 1 | 1 | 1 | 1 | NA |

**Appendix 3 table 2**: ORCHA score pairwise comparison - Tier B.

|  | **TB_1** | **TB_2** | **TB_3** | **TB_4** | **TB_5** | **TB_6** | **TB_7** | **TB_8** | **TB_9** | **TB_10** | **TB_11** | **TB_12** | **TB_13** | **TB_14** | **TB_15** | **TB_16** | **TB_17** | **TB_18** | **TB_19** | **TB_20** | **TB_21** | **TB_22** | **TB_23** | **TB_24** | **TB_25** | **TB_26** |
| --- | --- | --- | --- | --- | --- | --- | --- | --- | --- | --- | --- | --- | --- | --- | --- | --- | --- | --- | --- | --- | --- | --- | --- | --- | --- | --- |
| **TB_1** | NA |  |  |  |  |  |  |  |  |  |  |  |  |  |  |  |  |  |  |  |  |  |  |  |  |  |
| **TB_2** | 1 | NA |  |  |  |  |  |  |  |  |  |  |  |  |  |  |  |  |  |  |  |  |  |  |  |  |
| **TB_3** | .144 | 1 | NA |  |  |  |  |  |  |  |  |  |  |  |  |  |  |  |  |  |  |  |  |  |  |  |
| **TB_4** | .003 | .098 | 1 | NA |  |  |  |  |  |  |  |  |  |  |  |  |  |  |  |  |  |  |  |  |  |  |
| **TB_5** | .143 | 1 | 1 | 1 | NA |  |  |  |  |  |  |  |  |  |  |  |  |  |  |  |  |  |  |  |  |  |
| **TB_6** | .137 | 1 | 1 | 1 | 1 | NA |  |  |  |  |  |  |  |  |  |  |  |  |  |  |  |  |  |  |  |  |
| **TB_7** | <.001 | <.001 | 1 | 1 | 1 | 1 | NA |  |  |  |  |  |  |  |  |  |  |  |  |  |  |  |  |  |  |  |
| **TB_8** | 1 | 1 | 1 | 1 | 1 | 1 | 1 | NA |  |  |  |  |  |  |  |  |  |  |  |  |  |  |  |  |  |  |
| **TB_9** | .121 | .785 | 1 | 1 | 1 |  | 1 | 1 | NA |  |  |  |  |  |  |  |  |  |  |  |  |  |  |  |  |  |
| **TB_10** | <.001 | .001 | 1 | 1 | 1 | 1 | 1 | 1 | 1 | NA |  |  |  |  |  |  |  |  |  |  |  |  |  |  |  |  |
| **TB_11** | 1 | 1 | .185 | .020 | .105 | .083 | <.001 | .343 | .053 | <.001 | NA |  |  |  |  |  |  |  |  |  |  |  |  |  |  |  |
| **TB_12** | 1 | 1 | 1 | 1 | 1 | 1 | .540 | 1 | 1 | .752 | 1 | NA |  |  |  |  |  |  |  |  |  |  |  |  |  |  |
| **TB_13** | 1 | 1 | 1 | 1 | 1 | 1 | 1 | 1 | 1 | 1 | 1 | 1 | NA |  |  |  |  |  |  |  |  |  |  |  |  |  |
| **TB_14** | 1 | 1 | 1 | 1 | 1 | 1 | 1 | 1 | 1 | 1 | 1 | 1 | 1 | NA |  |  |  |  |  |  |  |  |  |  |  |  |
| **TB_15** | .004 | .001 | <.001 | <.001 | <.001 | <.001 | <.001 | <.001 | <.001 | <.001 | 1 | .019 | .001 | .002 | NA |  |  |  |  |  |  |  |  |  |  |  |
| **TB_16** | 1 | 1 | 1 | 1 | 1 | 1 | .118 | 1 | 1 | .172 | 1 | 1 | 1 | 1 | .464 | NA |  |  |  |  |  |  |  |  |  |  |
| **TB_17** | 1 | 1 | 1 | 1 | 1 | 1 | .707 | 1 | 1 | .839 | 1 | 1 | 1 | 1 | .635 | 1 | NA |  |  |  |  |  |  |  |  |  |
| **TB_18** | 1 | 1 | 1 | 1 | 1 | 1 | .034 | 1 | 1 | .047 | 1 | 1 | 1 | 1 | 1 | 1 | 1 | NA |  |  |  |  |  |  |  |  |
| **TB_19** | 1 | 1 | 1 | 1 | 1 | 1 | 1 | 1 | 1 | 1 | 1 | 1 | 1 | 1 | .172 | 1 | 1 | 1 | NA |  |  |  |  |  |  |  |
| **TB_20** | 1 | 1 | 1 | 1 | 1 | 1 | 1 | 1 | 1 | 1 | 1 | 1 | 1 | 1 | .064 | 1 | 1 | 1 | 1 | NA |  |  |  |  |  |  |
| **TB_21** | 1 | 1 | 1 | 1 | 1 | 1 | 1 | 1 | 1 | 1 | 1 | 1 | 1 | 1 | .008 | 1 | 1 | 1 | 1 | 1 | NA |  |  |  |  |  |
| **TB_22** | 1 | 1 | 1 | 1 | 1 | 1 | 1 | 1 | 1 | 1 | 1 | 1 | 1 | 1 | .007 | 1 | 1 | 1 | 1 | 1 | 1 | NA |  |  |  |  |
| **TB_23** | 1 | 1 | 1 | 1 | 1 | 1 | .752 | 1 | 1 | .829 | 1 | 1 | 1 | 1 | 1 | 1 | 1 | 1 | 1 | 1 | 1 | 1 | NA |  |  |  |
| **TB_24** | 1 | 1 | 1 | 1 | 1 | 1 | 1 | 1 | 1 | 1 | 1 | 1 | 1 | 1 | .013 | 1 | 1 | 1 | 1 | 1 | 1 | 1 | 1 | NA |  |  |
| **TB_25** | 1 | 1 | 1 | 1 | 1 | 1 | 1 | 1 | 1 | 1 | 1 | 1 | 1 | 1 | 1 | 1 | 1 | 1 | 1 | 1 | 1 | 1 | 1 | 1 | NA |  |
| **TB_26** | 1 | 1 | 1 | 1 | 1 | 1 | .972 | 1 | 1 | 1 | 1 | 1 | 1 | 1 | 1 | 1 | 1 | 1 | 1 | 1 | 1 | 1 | 1 | 1 | 1 | NA |

**Appendix 3 table 3**: ORCHA score pairwise comparison - Tier C.

|  | **TC_1** | **TC_2** | **TC_3** | **TC_4** | **TC_5** | **TC_6** | **TC_7** | **TC_8** | **TC_9** | **TC_10** | **TC_11** | **TC_12** | **TC_13** | **TC_14** | **TC_15** | **TC_16** | **TC_17** | **TC_18** | **TC_19** | **TC_20** | **TC_21** | **TC_22** | **TC_23** | **TC_24** | **TC_25** |
| --- | --- | --- | --- | --- | --- | --- | --- | --- | --- | --- | --- | --- | --- | --- | --- | --- | --- | --- | --- | --- | --- | --- | --- | --- | --- |
| **TC_1** | NA |  |  |  |  |  |  |  |  |  |  |  |  |  |  |  |  |  |  |  |  |  |  |  |  |
| **TC_2** | 1 | NA |  |  |  |  |  |  |  |  |  |  |  |  |  |  |  |  |  |  |  |  |  |  |  |
| **TC_3** | .876 | 1 | NA |  |  |  |  |  |  |  |  |  |  |  |  |  |  |  |  |  |  |  |  |  |  |
| **TC_4** | 1 | 1 | 1 | NA |  |  |  |  |  |  |  |  |  |  |  |  |  |  |  |  |  |  |  |  |  |
| **TC_5** | 1 | 1 | 1 | 1 | NA |  |  |  |  |  |  |  |  |  |  |  |  |  |  |  |  |  |  |  |  |
| **TC_6** | .199 | 1 | 1 | .489 | 1 | NA |  |  |  |  |  |  |  |  |  |  |  |  |  |  |  |  |  |  |  |
| **TC_7** | 1 | 1 | 1 | 1 | 1 | 1 | NA |  |  |  |  |  |  |  |  |  |  |  |  |  |  |  |  |  |  |
| **TC_8** | .797 | .007 | <.001 | 1 | .025 | <.001 | .442 | NA |  |  |  |  |  |  |  |  |  |  |  |  |  |  |  |  |  |
| **TC_9** | 1 | 1 | 1 | 1 | 1 | 1 | 1 | .033 | NA |  |  |  |  |  |  |  |  |  |  |  |  |  |  |  |  |
| **TC_10** | .001 | .163 | 1 | .004 | 1 | 1 | .528 | <.001 | 1 | NA |  |  |  |  |  |  |  |  |  |  |  |  |  |  |  |
| **TC_11** | 1 | 1 | .143 | 1 | 1 | .032 | 1 | 1 | 1 | <.001 | NA |  |  |  |  |  |  |  |  |  |  |  |  |  |  |
| **TC_12** | 1 | .096 | .009 | 1 | .131 | .002 | 1 | 1 | .146 | <.001 | 1 | NA |  |  |  |  |  |  |  |  |  |  |  |  |  |
| **TC_13** | 1 | 1 | 1 | 1 | 1 | 1 | 1 | 1 | 1 | .777 | 1 | 1 | NA |  |  |  |  |  |  |  |  |  |  |  |  |
| **TC_14** | 1 | 1 | 1 | 1 | 1 | 1 | 1 | 1 | 1 | 1 | 1 | 1 | 1 | NA |  |  |  |  |  |  |  |  |  |  |  |
| **TC_15** | .474 | 1 | 1 | .650 | 1 | 1 | 1 | .001 | 1 | 1 | .056 | .005 | 1 | 1 | NA |  |  |  |  |  |  |  |  |  |  |
| **TC_16** | 1 | 1 | 1 | 1 | 1 | .371 | 1 | 1 | 1 | .014 | 1 | 1 | 1 | 1 | .263 | NA |  |  |  |  |  |  |  |  |  |
| **TC_17** | 1 | 1 | 1 | 1 | 1 | 1 | 1 | 1 | 1 | 1 | 1 | 1 | 1 | 1 | 1 | 1 | NA |  |  |  |  |  |  |  |  |
| **TC_18** | 1 | 1 | 1 | 1 | 1 | 1 | 1 | 1 | 1 | 1 | 1 | 1 | 1 | 1 | 1 | 1 | 1 | NA |  |  |  |  |  |  |  |
| **TC_19** | 1 | 1 | 1 | 1 | 1 | 1 | 1 | .583 | 1 | 1 | 1 | .780 | 1 | 1 | 1 | 1 | 1 | 1 | NA |  |  |  |  |  |  |
| **TC_20** | 1 | 1 | 1 | 1 | 1 | 1 | 1 | 1 | 1 | .331 | 1 | 1 | 1 | 1 | 1 | 1 | 1 | 1 | 1 | NA |  |  |  |  |  |
| **TC_21** | 1 | 1 | 1 | 1 | 1 | 1 | 1 | .675 | 1 | 1 | 1 | .885 | 1 | 1 | 1 | 1 | 1 | 1 | 1 | 1 | NA |  |  |  |  |
| **TC_22** | 1 | 1 | 1 | 1 | 1 | .804 | 1 | 1 | 1 | .137 | 1 | 1 | 1 | 1 | .442 | 1 | 1 | 1 | 1 | 1 | 1 | NA |  |  |  |
| **TC_23** | 1 | 1 | 1 | 1 | 1 | 1 | 1 | .728 | 1 | 1 | 1 | .855 | 1 | 1 | 1 | 1 | 1 | 1 | 1 | 1 | 1 | 1 | NA |  |  |
| **TC_24** | 1 | 1 | 1 | 1 | 1 | 1 | 1 | 1 | 1 | 1 | 1 | 1 | 1 | 1 | 1 | 1 | 1 | 1 | 1 | 1 | 1 | 1 | 1 | NA |  |
| **TC_25** | 1 | 1 | 1 | 1 | 1 | 1 | 1 | 1 | 1 | 1 | 1 | 1 | 1 | 1 | 1 | 1 | 1 | 1 | 1 | 1 | 1 | 1 | 1 | 1 | NA |

**Appendix 3 table 4**: UX score pairwise comparison.

|  | **1** | **2** | **3** | **4** | **5** | **6** | **7** | **8** | **9** | **10** | **11** | **12** | **13** | **14** | **15** | **16** | **17** | **18** | **19** | **20** | **21** | **22** | **23** | **24** | **25** | **26** |
| --- | --- | --- | --- | --- | --- | --- | --- | --- | --- | --- | --- | --- | --- | --- | --- | --- | --- | --- | --- | --- | --- | --- | --- | --- | --- | --- |
| **1** | NA |  |  |  |  |  |  |  |  |  |  |  |  |  |  |  |  |  |  |  |  |  |  |  |  |  |
| **2** | 1 | NA |  |  |  |  |  |  |  |  |  |  |  |  |  |  |  |  |  |  |  |  |  |  |  |  |
| **3** | 1 | 1 | NA |  |  |  |  |  |  |  |  |  |  |  |  |  |  |  |  |  |  |  |  |  |  |  |
| **4** | 1 | 1 | 1 | NA |  |  |  |  |  |  |  |  |  |  |  |  |  |  |  |  |  |  |  |  |  |  |
| **5** | 1 | 1 | 1 | 1 | NA |  |  |  |  |  |  |  |  |  |  |  |  |  |  |  |  |  |  |  |  |  |
| **6** | .118 | .013 | .438 | 1 | 1 | NA |  |  |  |  |  |  |  |  |  |  |  |  |  |  |  |  |  |  |  |  |
| **7** | 1 | 1 | 1 | 1 | 1 | 1 | NA |  |  |  |  |  |  |  |  |  |  |  |  |  |  |  |  |  |  |  |
| **8** | 1 | 1 | 1 | 1 | 1 | 1 | 1 | NA |  |  |  |  |  |  |  |  |  |  |  |  |  |  |  |  |  |  |
| **9** | 1 | 1 | 1 | 1 | 1 | .002 | 1 | 1 | NA |  |  |  |  |  |  |  |  |  |  |  |  |  |  |  |  |  |
| **10** | 1 | 1 | 1 | 1 | 1 | .023 | 1 | 1 | 1 | NA |  |  |  |  |  |  |  |  |  |  |  |  |  |  |  |  |
| **11** | <.001 | <.001 | <.001 | <.001 | <.001 | <.001 | <.001 | .002 | 1 | .910 | NA |  |  |  |  |  |  |  |  |  |  |  |  |  |  |  |
| **12** | 1 | .415 | 1 | 1 | 1 | 1 | 1 | 1 | .030 | .185 | <.001 | NA |  |  |  |  |  |  |  |  |  |  |  |  |  |  |
| **13** | 1 | 1 | 1 | 1 | 1 | 1 | 1 | 1 | .978 | 1 | <.001 | 1 | NA |  |  |  |  |  |  |  |  |  |  |  |  |  |
| **14** | 1 | 1 | 1 | 1 | 1 | 1 | 1 | 1 | 1 | 1 | <.001 | 1 | 1 | NA |  |  |  |  |  |  |  |  |  |  |  |  |
| **15** | 1 | 1 | 1 | 1 | 1 | 1 | 1 | 1 | 1 | 1 | .013 | 1 | 1 | 1 | NA |  |  |  |  |  |  |  |  |  |  |  |
| **16** | 1 | 1 | 1 | 1 | 1 | 1 | 1 | 1 | 1 | 1 | .004 | 1 | 1 | 1 | 1 | NA |  |  |  |  |  |  |  |  |  |  |
| **17** | 1 | 1 | 1 | 1 | 1 | 1 | 1 | 1 | .934 | 1 | <.001 | 1 | 1 | 1 | 1 | 1 | NA |  |  |  |  |  |  |  |  |  |
| **18** | 1 | 1 | 1 | 1 | 1 | 1 | 1 | 1 | 1 | 1 | .953 | 1 | 1 | 1 | 1 | 1 | 1 | NA |  |  |  |  |  |  |  |  |
| **19** | 1 | 1 | 1 | 1 | 1 | .904 | 1 | 1 | 1 | 1 | 1 | 1 | 1 | 1 | 1 | 1 | 1 | 1 | NA |  |  |  |  |  |  |  |
| **20** | 1 | 1 | 1 | 1 | 1 | 1 | 1 | 1 | 1 | 1 | 1 | 1 | 1 | 1 | 1 | 1 | 1 | 1 | 1 | NA |  |  |  |  |  |  |
| **21** | 1 | 1 | 1 | 1 | 1 | .055 | 1 | 1 | 1 | 1 | 1 | .194 | 1 | 1 | 1 | 1 | 1 | 1 | 1 | 1 | NA |  |  |  |  |  |
| **22** | 1 | 1 | 1 | 1 | 1 | 1 | 1 | 1 | 1 | 1 | 1 | 1 | 1 | 1 | 1 | 1 | 1 | 1 | 1 | 1 | 1 | NA |  |  |  |  |
| **23** | 1 | 1 | 1 | 1 | 1 | 1 | 1 | 1 | 1 | 1 | .465 | 1 | 1 | 1 | 1 | 1 | 1 | 1 | 1 | 1 | 1 | 1 | NA |  |  |  |
| **24** | 1 | 1 | 1 | 1 | 1 | 1 | 1 | 1 | 1 | 1 | 1 | 1 | 1 | 1 | 1 | 1 | 1 | 1 | 1 | 1 | 1 | 1 | 1 | NA |  |  |
| **25** | 1 | 1 | 1 | 1 | 1 | 1 | 1 | 1 | 1 | 1 | .283 | 1 | 1 | 1 | 1 | 1 | 1 | 1 | 1 | 1 | 1 | 1 | 1 | 1 | NA |  |
| **26** | 1 | 1 | 1 | 1 | 1 | 1 | 1 | 1 | 1 | 1 | 1 | 1 | 1 | 1 | 1 | 1 | 1 | 1 | 1 | 1 | 1 | 1 | 1 | 1 | 1 | NA |

**Appendix 3 table 5**: UX score pairwise comparison - Tier B.

|  | **TB_1** | **TB_2** | **TB_3** | **TB_4** | **TB_5** | **TB_6** | **TB_7** | **TB_8** | **TB_9** | **TB_10** | **TB_11** | **TB_12** | **TB_13** | **TB_14** | **TB_15** | **TB_16** | **TB_17** | **TB_18** | **TB_19** | **TB_20** | **TB_21** | **TB_22** | **TB_23** | **TB_24** | **TB_25** | **TB_26** |
| --- | --- | --- | --- | --- | --- | --- | --- | --- | --- | --- | --- | --- | --- | --- | --- | --- | --- | --- | --- | --- | --- | --- | --- | --- | --- | --- |
| **TB_1** | NA |  |  |  |  |  |  |  |  |  |  |  |  |  |  |  |  |  |  |  |  |  |  |  |  |  |
| **TB_2** | 1 | NA |  |  |  |  |  |  |  |  |  |  |  |  |  |  |  |  |  |  |  |  |  |  |  |  |
| **TB_3** | 1 | 1 | NA |  |  |  |  |  |  |  |  |  |  |  |  |  |  |  |  |  |  |  |  |  |  |  |
| **TB_4** | 1 | 1 | 1 | NA |  |  |  |  |  |  |  |  |  |  |  |  |  |  |  |  |  |  |  |  |  |  |
| **TB_5** | 1 | 1 | 1 | 1 | NA |  |  |  |  |  |  |  |  |  |  |  |  |  |  |  |  |  |  |  |  |  |
| **TB_6** | 1 | 1 | 1 | 1 | 1 | NA |  |  |  |  |  |  |  |  |  |  |  |  |  |  |  |  |  |  |  |  |
| **TB_7** | 1 | 1 | 1 | 1 | 1 | 1 | NA |  |  |  |  |  |  |  |  |  |  |  |  |  |  |  |  |  |  |  |
| **TB_8** | 1 | 1 | 1 | 1 | 1 | 1 | 1 | NA |  |  |  |  |  |  |  |  |  |  |  |  |  |  |  |  |  |  |
| **TB_9** | 1 | 1 | 1 | 1 | 1 | 1 | 1 | 1 | NA |  |  |  |  |  |  |  |  |  |  |  |  |  |  |  |  |  |
| **TB_10** | 1 | 1 | 1 | 1 | 1 | 1 | 1 | 1 | 1 | NA |  |  |  |  |  |  |  |  |  |  |  |  |  |  |  |  |
| **TB_11** | 1 | 1 | 1 | 1 | 1 | .960 | 1 | 1 | 1 | .165 | NA |  |  |  |  |  |  |  |  |  |  |  |  |  |  |  |
| **TB_12** | 1 | 1 | 1 | 1 | 1 | 1 | 1 | 1 | 1 | 1 | 1 | NA |  |  |  |  |  |  |  |  |  |  |  |  |  |  |
| **TB_13** | 1 | 1 | 1 | 1 | 1 | 1 | 1 | 1 | 1 | 1 | .182 | 1 | NA |  |  |  |  |  |  |  |  |  |  |  |  |  |
| **TB_14** | 1 | 1 | 1 | 1 | 1 | 1 | 1 | 1 | 1 | 1 | 1 | 1 | 1 | NA |  |  |  |  |  |  |  |  |  |  |  |  |
| **TB_15** | .001 | .002 | .003 | .001 | .013 | <.001 | .009 | .074 | .001 | <.001 | 1 | .019 | <.001 | .002 | NA |  |  |  |  |  |  |  |  |  |  |  |
| **TB_16** | 1 | 1 | 1 | 1 | 1 | 1 | 1 | 1 | 1 | 1 | 1 | 1 | 1 | 1 | .338 | NA |  |  |  |  |  |  |  |  |  |  |
| **TB_17** | 1 | 1 | 1 | 1 | 1 | 1 | 1 | 1 | 1 | 1 | 1 | 1 | 1 | 1 | 1 | 1 | NA |  |  |  |  |  |  |  |  |  |
| **TB_18** | 1 | 1 | 1 | 1 | 1 | 1 | 1 | 1 | 1 | .217 | 1 | 1 | .221 | 1 | 1 | 1 | 1 | NA |  |  |  |  |  |  |  |  |
| **TB_19** | 1 | 1 | 1 | 1 | 1 | 1 | 1 | 1 | 1 | 1 | 1 | 1 | 1 | 1 | .928 | 1 | 1 | 1 | NA |  |  |  |  |  |  |  |
| **TB_20** | 1 | 1 | 1 | 1 | 1 | 1 | 1 | 1 | 1 | 1 | 1 | 1 | 1 | 1 | .206 | 1 | 1 | 1 | 1 | NA |  |  |  |  |  |  |
| **TB_21** | 1 | 1 | 1 | 1 | 1 | 1 | 1 | 1 | 1 | 1 | .513 | 1 | 1 | 1 | .001 | 1 | 1 | .378 | 1 | 1 | NA |  |  |  |  |  |
| **TB_22** | 1 | 1 | 1 | 1 | 1 | 1 | 1 | 1 | 1 | 1 | 1 | 1 | 1 | 1 | 1 | 1 | 1 | 1 | 1 | 1 | 1 | NA |  |  |  |  |
| **TB_23** | 1 | 1 | 1 | 1 | 1 | 1 | 1 | 1 | 1 | 1 | 1 | 1 | 1 | 1 | 1 | 1 | 1 | 1 | 1 | 1 | 1 | 1 | NA |  |  |  |
| **TB_24** | 1 | 1 | 1 | 1 | 1 | 1 | 1 | 1 | 1 | 1 | 1 | 1 | 1 | 1 | .102 | 1 | 1 | 1 | 1 | 1 | 1 | 1 | 1 | NA |  |  |
| **TB_25** | 1 | 1 | 1 | 1 | 1 | 1 | 1 | 1 | 1 | 1 | 1 | 1 | 1 | 1 | 1 | 1 | 1 | 1 | 1 | 1 | 1 | 1 | 1 | 1 | NA |  |
| **TB_26** | 1 | 1 | 1 | 1 | 1 | 1 | 1 | 1 | 1 | 1 | 1 | 1 | 1 | 1 | 1 | 1 | 1 | 1 | 1 | 1 | 1 | 1 | 1 | 1 | 1 | NA |

**Appendix 3 table 6**: UX score pairwise comparison - Tier C.

|  | **TC_1** | **TC_2** | **TC_3** | **TC_4** | **TC_5** | **TC_6** | **TC_7** | **TC_8** | **TC_9** | **TC_10** | **TC_11** | **TC_12** | **TC_13** | **TC_14** | **TC_15** | **TC_16** | **TC_17** | **TC_18** | **TC_19** | **TC_20** | **TC_21** | **TC_22** | **TC_23** | **TC_24** | **TC_25** |
| --- | --- | --- | --- | --- | --- | --- | --- | --- | --- | --- | --- | --- | --- | --- | --- | --- | --- | --- | --- | --- | --- | --- | --- | --- | --- |
| **TC_1** | NA |  |  |  |  |  |  |  |  |  |  |  |  |  |  |  |  |  |  |  |  |  |  |  |  |
| **TC_2** | 1 | NA |  |  |  |  |  |  |  |  |  |  |  |  |  |  |  |  |  |  |  |  |  |  |  |
| **TC_3** | 1 | 1 | NA |  |  |  |  |  |  |  |  |  |  |  |  |  |  |  |  |  |  |  |  |  |  |
| **TC_4** | .660 | .014 | .589 | NA |  |  |  |  |  |  |  |  |  |  |  |  |  |  |  |  |  |  |  |  |  |
| **TC_5** | 1 | 1 | 1 | 1 | NA |  |  |  |  |  |  |  |  |  |  |  |  |  |  |  |  |  |  |  |  |
| **TC_6** | 1 | 1 | 1 | .089 | 1 | NA |  |  |  |  |  |  |  |  |  |  |  |  |  |  |  |  |  |  |  |
| **TC_7** | 1 | 1 | 1 | 1 | 1 | 1 | NA |  |  |  |  |  |  |  |  |  |  |  |  |  |  |  |  |  |  |
| **TC_8** | 1 | 1 | 1 | 1 | 1 | 1 | 1 | NA |  |  |  |  |  |  |  |  |  |  |  |  |  |  |  |  |  |
| **TC_9** | 1 | 1 | 1 | 1 | 1 | 1 | 1 | 1 | NA |  |  |  |  |  |  |  |  |  |  |  |  |  |  |  |  |
| **TC_10** | 1 | 1 | 1 | .001 | 1 | 1 | 1 | 1 | 1 | NA |  |  |  |  |  |  |  |  |  |  |  |  |  |  |  |
| **TC_11** | 1 | 1 | 1 | 1 | 1 | 1 | 1 | 1 | 1 | 1 | NA |  |  |  |  |  |  |  |  |  |  |  |  |  |  |
| **TC_12** | 1 | 1 | 1 | 1 | 1 | 1 | 1 | 1 | 1 | 1 | 1 | NA |  |  |  |  |  |  |  |  |  |  |  |  |  |
| **TC_13** | 1 | 1 | 1 | 1 | 1 | 1 | 1 | 1 | 1 | 1 | 1 | 1 | NA |  |  |  |  |  |  |  |  |  |  |  |  |
| **TC_14** | .864 | .096 | .562 | 1 | 1 | .110 | 1 | 1 | .685 | .003 | 1 | 1 | 1 | NA |  |  |  |  |  |  |  |  |  |  |  |
| **TC_15** | 1 | 1 | 1 | 1 | 1 | 1 | 1 | 1 | 1 | 1 | 1 | 1 | 1 | .497 | NA |  |  |  |  |  |  |  |  |  |  |
| **TC_16** | 1 | 1 | 1 | 1 | 1 | 1 | 1 | 1 | 1 | 1 | 1 | 1 | 1 | 1 | 1 | NA |  |  |  |  |  |  |  |  |  |
| **TC_17** | 1 | 1 | 1 | 1 | 1 | 1 | 1 | 1 | 1 | 1 | 1 | 1 | 1 | 1 | 1 | 1 | NA |  |  |  |  |  |  |  |  |
| **TC_18** | 1 | 1 | 1 | 1 | 1 | 1 | 1 | 1 | 1 | 1 | 1 | 1 | 1 | 1 | 1 | 1 | 1 | NA |  |  |  |  |  |  |  |
| **TC_19** | 1 | 1 | 1 | 1 | 1 | 1 | 1 | 1 | 1 | 1 | 1 | 1 | 1 | 1 | 1 | 1 | 1 | 1 | NA |  |  |  |  |  |  |
| **TC_20** | 1 | 1 | 1 | 1 | 1 | 1 | 1 | 1 | 1 | 1 | 1 | 1 | 1 | 1 | 1 | 1 | 1 | 1 | 1 | NA |  |  |  |  |  |
| **TC_21** | 1 | 1 | 1 | 1 | 1 | 1 | 1 | 1 | 1 | 1 | 1 | 1 | 1 | 1 | 1 | 1 | 1 | 1 | 1 | 1 | NA |  |  |  |  |
| **TC_22** | 1 | 1 | 1 | 1 | 1 | 1 | 1 | 1 | 1 | 1 | 1 | 1 | 1 | 1 | 1 | 1 | 1 | 1 | 1 | 1 | 1 | NA |  |  |  |
| **TC_23** | 1 | 1 | 1 | 1 | 1 | 1 | 1 | 1 | 1 | 1 | 1 | 1 | 1 | 1 | 1 | 1 | 1 | 1 | 1 | 1 | 1 | 1 | NA |  |  |
| **TC_24** | 1 | 1 | 1 | 1 | 1 | 1 | 1 | 1 | 1 | 1 | 1 | 1 | 1 | .908 | 1 | 1 | 1 | 1 | 1 | 1 | 1 | 1 | 1 | NA |  |
| **TC_25** | 1 | 1 | 1 | 1 | 1 | 1 | 1 | 1 | 1 | 1 | 1 | 1 | 1 | 1 | 1 | 1 | 1 | 1 | 1 | 1 | 1 | 1 | 1 | 1 | NA |

**Appendix 3 table 7**: PCA score pairwise comparison.

|  | **1** | **2** | **3** | **4** | **5** | **6** | **7** | **8** | **9** | **10** | **11** | **12** | **13** | **14** | **15** | **16** | **17** | **18** | **19** | **20** | **21** | **22** | **23** | **24** | **25** | **26** |
| --- | --- | --- | --- | --- | --- | --- | --- | --- | --- | --- | --- | --- | --- | --- | --- | --- | --- | --- | --- | --- | --- | --- | --- | --- | --- | --- |
| **1** | NA |  |  |  |  |  |  |  |  |  |  |  |  |  |  |  |  |  |  |  |  |  |  |  |  |  |
| **2** | 1 | NA |  |  |  |  |  |  |  |  |  |  |  |  |  |  |  |  |  |  |  |  |  |  |  |  |
| **3** | 1 | 1 | NA |  |  |  |  |  |  |  |  |  |  |  |  |  |  |  |  |  |  |  |  |  |  |  |
| **4** | .050 | .423 | 1 | NA |  |  |  |  |  |  |  |  |  |  |  |  |  |  |  |  |  |  |  |  |  |  |
| **5** | 1 | 1 | 1 | 1 | NA |  |  |  |  |  |  |  |  |  |  |  |  |  |  |  |  |  |  |  |  |  |
| **6** | 1 | 1 | 1 | 1 | 1 | NA |  |  |  |  |  |  |  |  |  |  |  |  |  |  |  |  |  |  |  |  |
| **7** | <.001 | <.001 | .006 | .103 | .002 | .046 | NA |  |  |  |  |  |  |  |  |  |  |  |  |  |  |  |  |  |  |  |
| **8** | .008 | .050 | 1 | 1 | 1 | 1 | 1 | NA |  |  |  |  |  |  |  |  |  |  |  |  |  |  |  |  |  |  |
| **9** | 1 | 1 | .144 | .016 | 1 | .529 | <.001 | .002 | NA |  |  |  |  |  |  |  |  |  |  |  |  |  |  |  |  |  |
| **10** | 1 | 1 | .020 | .002 | .874 | .096 | <.001 | <.001 | 1 | NA |  |  |  |  |  |  |  |  |  |  |  |  |  |  |  |  |
| **11** | <.001 | <.001 | <.001 | <.001 | <.001 | <.001 | <.001 | <.001 | 1 | 1 | NA |  |  |  |  |  |  |  |  |  |  |  |  |  |  |  |
| **12** | 1 | 1 | 1 | 1 | 1 | 1 | 1 | 1 | .210 | .040 | <.001 | NA |  |  |  |  |  |  |  |  |  |  |  |  |  |  |
| **13** | .004 | .021 | 1 | 1 | 1 | 1 | 1 | 1 | .001 | <.001 | <.001 | 1 | NA |  |  |  |  |  |  |  |  |  |  |  |  |  |
| **14** | 1 | 1 | 1 | 1 | 1 | 1 | 1 | 1 | .123 | .022 | <.001 | 1 | 1 | NA |  |  |  |  |  |  |  |  |  |  |  |  |
| **15** | .900 | 1 | 1 | 1 | 1 | 1 | 1 | 1 | .083 | .015 | <.001 | 1 | 1 | 1 | NA |  |  |  |  |  |  |  |  |  |  |  |
| **16** | 1 | 1 | 1 | 1 | 1 | 1 | .034 | 1 | 1 | 1 | .012 | 1 | 1 | 1 | 1 | NA |  |  |  |  |  |  |  |  |  |  |
| **17** | .043 | .133 | 1 | 1 | 1 | 1 | 1 | 1 | .004 | .001 | <.001 | 1 | 1 | 1 | 1 | 1 | NA |  |  |  |  |  |  |  |  |  |
| **18** | 1 | 1 | 1 | 1 | 1 | 1 | .013 | 1 | 1 | 1 | .948 | 1 | 1 | 1 | 1 | 1 | 1 | NA |  |  |  |  |  |  |  |  |
| **19** | 1 | 1 | 1 | 1 | 1 | 1 | .444 | 1 | 1 | 1 | .147 | 1 | 1 | 1 | 1 | 1 | 1 | 1 | NA |  |  |  |  |  |  |  |
| **20** | 1 | 1 | 1 | 1 | 1 | 1 | .900 | 1 | 1 | 1 | .098 | 1 | 1 | 1 | 1 | 1 | 1 | 1 | 1 | NA |  |  |  |  |  |  |
| **21** | 1 | 1 | 1 | 1 | 1 | 1 | .050 | 1 | 1 | 1 | 1 | 1 | 1 | 1 | 1 | 1 | 1 | 1 | 1 | 1 | NA |  |  |  |  |  |
| **22** | 1 | 1 | 1 | 1 | 1 | 1 | 1 | 1 | 1 | 1 | .449 | 1 | 1 | 1 | 1 | 1 | 1 | 1 | 1 | 1 | 1 | NA |  |  |  |  |
| **23** | 1 | 1 | 1 | 1 | 1 | 1 | 1 | 1 | .160 | .050 | <.001 | 1 | 1 | 1 | 1 | 1 | 1 | 1 | 1 | 1 | 1 | 1 | NA |  |  |  |
| **24** | 1 | 1 | 1 | 1 | 1 | 1 | 1 | 1 | .501 | .168 | .002 | 1 | 1 | 1 | 1 | 1 | 1 | 1 | 1 | 1 | 1 | 1 | 1 | NA |  |  |
| **25** | 1 | 1 | 1 | 1 | 1 | 1 | .112 | 1 | 1 | 1 | 1 | 1 | 1 | 1 | 1 | 1 | 1 | 1 | 1 | 1 | 1 | 1 | 1 | 1 | NA |  |
| **26** | 1 | 1 | 1 | 1 | 1 | 1 | 1 | 1 | 1 | 1 | .028 | 1 | 1 | 1 | 1 | 1 | 1 | 1 | 1 | 1 | 1 | 1 | 1 | 1 | 1 | NA |

**Appendix 3 table 8**: PCA score pairwise comparison - Tier B.

|  | **TB_1** | **TB_2** | **TB_3** | **TB_4** | **TB_5** | **TB_6** | **TB_7** | **TB_8** | **TB_9** | **TB_10** | **TB_11** | **TB_12** | **TB_13** | **TB_14** | **TB_15** | **TB_16** | **TB_17** | **TB_18** | **TB_19** | **TB_20** | **TB_21** | **TB_22** | **TB_23** | **TB_24** | **TB_25** | **TB_26** |
| --- | --- | --- | --- | --- | --- | --- | --- | --- | --- | --- | --- | --- | --- | --- | --- | --- | --- | --- | --- | --- | --- | --- | --- | --- | --- | --- |
| **TB_1** | NA |  |  |  |  |  |  |  |  |  |  |  |  |  |  |  |  |  |  |  |  |  |  |  |  |  |
| **TB_2** | 1 | NA |  |  |  |  |  |  |  |  |  |  |  |  |  |  |  |  |  |  |  |  |  |  |  |  |
| **TB_3** | .001 | .099 | NA |  |  |  |  |  |  |  |  |  |  |  |  |  |  |  |  |  |  |  |  |  |  |  |
| **TB_4** | <.001 | .002 | 1 | NA |  |  |  |  |  |  |  |  |  |  |  |  |  |  |  |  |  |  |  |  |  |  |
| **TB_5** | .025 | .614 | 1 | 1 | NA |  |  |  |  |  |  |  |  |  |  |  |  |  |  |  |  |  |  |  |  |  |
| **TB_6** | .170 | 1 | 1 | 1 | 1 | NA |  |  |  |  |  |  |  |  |  |  |  |  |  |  |  |  |  |  |  |  |
| **TB_7** | <.001 | <.001 | 1 | 1 | 1 | 1 | NA |  |  |  |  |  |  |  |  |  |  |  |  |  |  |  |  |  |  |  |
| **TB_8** | .107 | 1 | 1 | 1 | 1 | 1 | 1 | NA |  |  |  |  |  |  |  |  |  |  |  |  |  |  |  |  |  |  |
| **TB_9** | .031 | .420 | 1 | 1 | 1 | 1 | 1 | 1 | NA |  |  |  |  |  |  |  |  |  |  |  |  |  |  |  |  |  |
| **TB_10** | <.001 | <.001 | 1 | 1 | 1 | 1 | 1 | 1 | 1 | NA |  |  |  |  |  |  |  |  |  |  |  |  |  |  |  |  |
| **TB_11** | 1 | 1 | .731 | .100 | 1 | 1 | .001 | 1 | .566 | .003 | NA |  |  |  |  |  |  |  |  |  |  |  |  |  |  |  |
| **TB_12** | 1 | 1 | 1 | 1 | 1 | 1 | .476 | 1 | 1 | .657 | 1 | NA |  |  |  |  |  |  |  |  |  |  |  |  |  |  |
| **TB_13** | 1 | 1 | 1 | 1 | 1 | 1 | 1 | 1 | 1 | 1 | 1 | 1 | NA |  |  |  |  |  |  |  |  |  |  |  |  |  |
| **TB_14** | 1 | 1 | 1 | 1 | 1 | 1 | 1 | 1 | 1 | 1 | 1 | 1 | 1 | NA |  |  |  |  |  |  |  |  |  |  |  |  |
| **TB_15** | .478 | .071 | <.001 | <.001 | <.001 | .001 | <.001 | <.001 | <.001 | <.001 | 1 | .232 | .024 | .020 | NA |  |  |  |  |  |  |  |  |  |  |  |
| **TB_16** | 1 | 1 | 1 | 1 | 1 | 1 | .107 | 1 | 1 | .158 | 1 | 1 | 1 | 1 | 1 | NA |  |  |  |  |  |  |  |  |  |  |
| **TB_17** | 1 | 1 | 1 | 1 | 1 | 1 | 1 | 1 | 1 | 1 | 1 | 1 | 1 | 1 | .352 | 1 | NA |  |  |  |  |  |  |  |  |  |
| **TB_18** | 1 | 1 | 1 | 1 | 1 | 1 | .178 | 1 | 1 | .221 | 1 | 1 | 1 | 1 | 1 | 1 | 1 | NA |  |  |  |  |  |  |  |  |
| **TB_19** | 1 | 1 | 1 | 1 | 1 | 1 | 1 | 1 | 1 | 1 | 1 | 1 | 1 | 1 | 1 | 1 | 1 | 1 | NA |  |  |  |  |  |  |  |
| **TB_20** | 1 | 1 | 1 | 1 | 1 | 1 | 1 | 1 | 1 | 1 | 1 | 1 | 1 | 1 | .060 | 1 | 1 | 1 | 1 | NA |  |  |  |  |  |  |
| **TB_21** | 1 | 1 | 1 | 1 | 1 | 1 | 1 | 1 | 1 | 1 | 1 | 1 | 1 | 1 | .049 | 1 | 1 | 1 | 1 | 1 | NA |  |  |  |  |  |
| **TB_22** | 1 | 1 | 1 | 1 | 1 | 1 | 1 | 1 | 1 | 1 | 1 | 1 | 1 | 1 | .014 | 1 | 1 | 1 | 1 | 1 | 1 | NA |  |  |  |  |
| **TB_23** | 1 | 1 | 1 | 1 | 1 | 1 | 1 | 1 | 1 | 1 | 1 | 1 | 1 | 1 | 1 | 1 | 1 | 1 | 1 | 1 | 1 | 1 | NA |  |  |  |
| **TB_24** | 1 | 1 | 1 | 1 | 1 | 1 | 1 | 1 | 1 | 1 | 1 | 1 | 1 | 1 | .318 | 1 | 1 | 1 | 1 | 1 | 1 | 1 | 1 | NA |  |  |
| **TB_25** | 1 | 1 | 1 | 1 | 1 | 1 | 1 | 1 | 1 | 1 | 1 | 1 | 1 | 1 | .508 | 1 | 1 | 1 | 1 | 1 | 1 | 1 | 1 | 1 | NA |  |
| **TB_26** | 1 | 1 | 1 | 1 | 1 | 1 | 1 | 1 | 1 | 1 | 1 | 1 | 1 | 1 | 1 | 1 | 1 | 1 | 1 | 1 | 1 | 1 | 1 | 1 | 1 | NA |

**Appendix 3 table 9**: PCA score pairwise comparison - Tier C.

|  | **TC_1** | **TC_2** | **TC_3** | **TC_4** | **TC_5** | **TC_6** | **TC_7** | **TC_8** | **TC_9** | **TC_10** | **TC_11** | **TC_12** | **TC_13** | **TC_14** | **TC_15** | **TC_16** | **TC_17** | **TC_18** | **TC_19** | **TC_20** | **TC_21** | **TC_22** | **TC_23** | **TC_24** | **TC_25** |
| --- | --- | --- | --- | --- | --- | --- | --- | --- | --- | --- | --- | --- | --- | --- | --- | --- | --- | --- | --- | --- | --- | --- | --- | --- | --- |
| **TC_1** | NA |  |  |  |  |  |  |  |  |  |  |  |  |  |  |  |  |  |  |  |  |  |  |  |  |
| **TC_2** | 1 | NA |  |  |  |  |  |  |  |  |  |  |  |  |  |  |  |  |  |  |  |  |  |  |  |
| **TC_3** | .919 | 1 | NA |  |  |  |  |  |  |  |  |  |  |  |  |  |  |  |  |  |  |  |  |  |  |
| **TC_4** | 1 | 1 | 1 | NA |  |  |  |  |  |  |  |  |  |  |  |  |  |  |  |  |  |  |  |  |  |
| **TC_5** | 1 | 1 | 1 | 1 | NA |  |  |  |  |  |  |  |  |  |  |  |  |  |  |  |  |  |  |  |  |
| **TC_6** | .001 | .131 | 1 | .010 | 1 | NA |  |  |  |  |  |  |  |  |  |  |  |  |  |  |  |  |  |  |  |
| **TC_7** | 1 | 1 | 1 | 1 | 1 | .737 | NA |  |  |  |  |  |  |  |  |  |  |  |  |  |  |  |  |  |  |
| **TC_8** | .720 | .015 | <.001 | 1 | .001 | <.001 | .501 | NA |  |  |  |  |  |  |  |  |  |  |  |  |  |  |  |  |  |
| **TC_9** | .299 | 1 | 1 | .777 | 1 | 1 | 1 | <.001 | NA |  |  |  |  |  |  |  |  |  |  |  |  |  |  |  |  |
| **TC_10** | .001 | .073 | 1 | .006 | 1 | 1 | .412 | <.001 | 1 | NA |  |  |  |  |  |  |  |  |  |  |  |  |  |  |  |
| **TC_11** | 1 | 1 | 1 | 1 | 1 | .009 | 1 | 1 | .358 | .005 | NA |  |  |  |  |  |  |  |  |  |  |  |  |  |  |
| **TC_12** | 1 | .220 | .011 | 1 | .013 | <.001 | 1 | 1 | .003 | <.001 | 1 | NA |  |  |  |  |  |  |  |  |  |  |  |  |  |
| **TC_13** | 1 | 1 | 1 | 1 | 1 | 1 | 1 | 1 | 1 | 1 | 1 | 1 | NA |  |  |  |  |  |  |  |  |  |  |  |  |
| **TC_14** | 1 | 1 | 1 | 1 | 1 | 1 | 1 | .684 | 1 | 1 | 1 | 1 | 1 | NA |  |  |  |  |  |  |  |  |  |  |  |
| **TC_15** | .261 | 1 | 1 | .468 | 1 | 1 | 1 | .001 | 1 | 1 | .195 | .003 | 1 | 1 | NA |  |  |  |  |  |  |  |  |  |  |
| **TC_16** | 1 | 1 | 1 | 1 | 1 | .472 | 1 | 1 | 1 | .277 | 1 | 1 | 1 | 1 | 1 | NA |  |  |  |  |  |  |  |  |  |
| **TC_17** | 1 | 1 | 1 | 1 | 1 | 1 | 1 | 1 | 1 | 1 | 1 | 1 | 1 | 1 | 1 | 1 | NA |  |  |  |  |  |  |  |  |
| **TC_18** | 1 | 1 | 1 | 1 | 1 | 1 | 1 | 1 | 1 | 1 | 1 | 1 | 1 | 1 | 1 | 1 | 1 | NA |  |  |  |  |  |  |  |
| **TC_19** | 1 | 1 | 1 | 1 | 1 | 1 | 1 | .675 | 1 | 1 | 1 | .990 | 1 | 1 | 1 | 1 | 1 | 1 | NA |  |  |  |  |  |  |
| **TC_20** | 1 | 1 | 1 | 1 | 1 | .239 | 1 | 1 | 1 | .150 | 1 | 1 | 1 | 1 | .512 | 1 | 1 | 1 | 1 | NA |  |  |  |  |  |
| **TC_21** | 1 | 1 | 1 | 1 | 1 | 1 | 1 | .453 | 1 | 1 | 1 | .689 | 1 | 1 | 1 | 1 | 1 | 1 | 1 | 1 | NA |  |  |  |  |
| **TC_22** | 1 | 1 | .748 | 1 | .599 | .071 | 1 | 1 | .312 | .047 | 1 | 1 | 1 | 1 | .132 | 1 | 1 | 1 | 1 | 1 | .800 | NA |  |  |  |
| **TC_23** | 1 | 1 | 1 | 1 | 1 | 1 | 1 | 1 | 1 | 1 | 1 | 1 | 1 | 1 | 1 | 1 | 1 | 1 | 1 | 1 | 1 | 1 | NA |  |  |
| **TC_24** | 1 | 1 | 1 | 1 | 1 | 1 | 1 | 1 | 1 | 1 | 1 | 1 | 1 | 1 | 1 | 1 | 1 | 1 | 1 | 1 | 1 | 1 | 1 | NA |  |
| **TC_25** | 1 | 1 | 1 | 1 | 1 | 1 | 1 | 1 | 1 | 1 | 1 | 1 | 1 | 1 | 1 | 1 | 1 | 1 | 1 | 1 | 1 | 1 | 1 | 1 | NA |

**Appendix 3 table 10**: DP score pairwise comparison.

|  | **1** | **2** | **3** | **4** | **5** | **6** | **7** | **8** | **9** | **10** | **11** | **12** | **13** | **14** | **15** | **16** | **17** | **18** | **19** | **20** | **21** | **22** | **23** | **24** | **25** | **26** |
| --- | --- | --- | --- | --- | --- | --- | --- | --- | --- | --- | --- | --- | --- | --- | --- | --- | --- | --- | --- | --- | --- | --- | --- | --- | --- | --- |
| **1** | NA |  |  |  |  |  |  |  |  |  |  |  |  |  |  |  |  |  |  |  |  |  |  |  |  |  |
| **2** | 1 | NA |  |  |  |  |  |  |  |  |  |  |  |  |  |  |  |  |  |  |  |  |  |  |  |  |
| **3** | 1 | 1 | NA |  |  |  |  |  |  |  |  |  |  |  |  |  |  |  |  |  |  |  |  |  |  |  |
| **4** | 1 | 1 | 1 | NA |  |  |  |  |  |  |  |  |  |  |  |  |  |  |  |  |  |  |  |  |  |  |
| **5** | 1 | 1 | 1 | 1 | NA |  |  |  |  |  |  |  |  |  |  |  |  |  |  |  |  |  |  |  |  |  |
| **6** | 1 | 1 | 1 | 1 | 1 | NA |  |  |  |  |  |  |  |  |  |  |  |  |  |  |  |  |  |  |  |  |
| **7** | .029 | .035 | 1 | .021 | 1 | 1 | NA |  |  |  |  |  |  |  |  |  |  |  |  |  |  |  |  |  |  |  |
| **8** | 1 | 1 | 1 | 1 | 1 | 1 | 1 | NA |  |  |  |  |  |  |  |  |  |  |  |  |  |  |  |  |  |  |
| **9** | 1 | 1 | 1 | 1 | 1 | 1 | .730 | 1 | NA |  |  |  |  |  |  |  |  |  |  |  |  |  |  |  |  |  |
| **10** | 1 | 1 | 1 | 1 | 1 | 1 | 1 | 1 | 1 | NA |  |  |  |  |  |  |  |  |  |  |  |  |  |  |  |  |
| **11** | .223 | .264 | .054 | 1 | .006 | .179 | <.001 | 1 | 1 | 1 | NA |  |  |  |  |  |  |  |  |  |  |  |  |  |  |  |
| **12** | 1 | 1 | 1 | 1 | 1 | 1 | 1 | 1 | 1 | 1 | .047 | NA |  |  |  |  |  |  |  |  |  |  |  |  |  |  |
| **13** | 1 | 1 | 1 | 1 | 1 | 1 | 1 | 1 | 1 | 1 | .003 | 1 | NA |  |  |  |  |  |  |  |  |  |  |  |  |  |
| **14** | 1 | 1 | 1 | 1 | 1 | 1 | 1 | 1 | 1 | 1 | .004 | 1 | 1 | NA |  |  |  |  |  |  |  |  |  |  |  |  |
| **15** | 1 | 1 | 1 | 1 | 1 | 1 | 1 | 1 | 1 | 1 | 1 | 1 | 1 | 1 | NA |  |  |  |  |  |  |  |  |  |  |  |
| **16** | 1 | 1 | 1 | 1 | 1 | 1 | 1 | 1 | 1 | 1 | 1 | 1 | 1 | 1 | 1 | NA |  |  |  |  |  |  |  |  |  |  |
| **17** | 1 | 1 | 1 | 1 | 1 | 1 | 1 | 1 | 1 | 1 | .251 | 1 | 1 | 1 | 1 | 1 | NA |  |  |  |  |  |  |  |  |  |
| **18** | 1 | 1 | 1 | 1 | 1 | 1 | .174 | 1 | 1 | 1 | 1 | 1 | 1 | 1 | 1 | 1 | 1 | NA |  |  |  |  |  |  |  |  |
| **19** | 1 | 1 | 1 | 1 | .288 | 1 | .009 | 1 | 1 | 1 | 1 | .697 | .104 | .130 | 1 | 1 | 1 | 1 | NA |  |  |  |  |  |  |  |
| **20** | 1 | 1 | 1 | 1 | 1 | 1 | 1 | 1 | 1 | 1 | .058 | 1 | 1 | 1 | 1 | 1 | 1 | 1 | .357 | NA |  |  |  |  |  |  |
| **21** | 1 | 1 | 1 | 1 | 1 | 1 | 1 | 1 | 1 | 1 | 1 | 1 | 1 | 1 | 1 | 1 | 1 | 1 | 1 | 1 | NA |  |  |  |  |  |
| **22** | 1 | 1 | 1 | 1 | 1 | 1 | 1 | 1 | 1 | 1 | 1 | 1 | 1 | 1 | 1 | 1 | 1 | 1 | 1 | 1 | 1 | NA |  |  |  |  |
| **23** | 1 | 1 | 1 | 1 | 1 | 1 | 1 | 1 | 1 | 1 | 1 | 1 | 1 | 1 | 1 | 1 | 1 | 1 | 1 | 1 | 1 | 1 | NA |  |  |  |
| **24** | 1 | 1 | 1 | 1 | 1 | 1 | 1 | 1 | 1 | 1 | 1 | 1 | 1 | 1 | 1 | 1 | 1 | 1 | 1 | 1 | 1 | 1 | 1 | NA |  |  |
| **25** | 1 | 1 | 1 | 1 | 1 | 1 | .111 | 1 | 1 | 1 | 1 | 1 | .524 | .609 | 1 | 1 | 1 | 1 | 1 | .888 | 1 | 1 | 1 | 1 | NA |  |
| **26** | 1 | 1 | 1 | 1 | 1 | 1 | 1 | 1 | 1 | 1 | 1 | 1 | 1 | 1 | 1 | 1 | 1 | 1 | 1 | 1 | 1 | 1 | 1 | 1 | 1 | NA |

**Appendix 3 table 11**: DP score pairwise comparison - Tier B.

|  | **TB_1** | **TB_2** | **TB_3** | **TB_4** | **TB_5** | **TB_6** | **TB_7** | **TB_8** | **TB_9** | **TB_10** | **TB_11** | **TB_12** | **TB_13** | **TB_14** | **TB_15** | **TB_16** | **TB_17** | **TB_18** | **TB_19** | **TB_20** | **TB_21** | **TB_22** | **TB_23** | **TB_24** | **TB_25** | **TB_26** |
| --- | --- | --- | --- | --- | --- | --- | --- | --- | --- | --- | --- | --- | --- | --- | --- | --- | --- | --- | --- | --- | --- | --- | --- | --- | --- | --- |
| **TB_1** | NA |  |  |  |  |  |  |  |  |  |  |  |  |  |  |  |  |  |  |  |  |  |  |  |  |  |
| **TB_2** | 1 | NA |  |  |  |  |  |  |  |  |  |  |  |  |  |  |  |  |  |  |  |  |  |  |  |  |
| **TB_3** | 1 | 1 | NA |  |  |  |  |  |  |  |  |  |  |  |  |  |  |  |  |  |  |  |  |  |  |  |
| **TB_4** | 1 | 1 | 1 | NA |  |  |  |  |  |  |  |  |  |  |  |  |  |  |  |  |  |  |  |  |  |  |
| **TB_5** | 1 | 1 | 1 | 1 | NA |  |  |  |  |  |  |  |  |  |  |  |  |  |  |  |  |  |  |  |  |  |
| **TB_6** | 1 | 1 | 1 | 1 | 1 | NA |  |  |  |  |  |  |  |  |  |  |  |  |  |  |  |  |  |  |  |  |
| **TB_7** | .038 | .096 | .021 | 1 | 1 | 1 | NA |  |  |  |  |  |  |  |  |  |  |  |  |  |  |  |  |  |  |  |
| **TB_8** | 1 | 1 | 1 | 1 | 1 | 1 | 1 | NA |  |  |  |  |  |  |  |  |  |  |  |  |  |  |  |  |  |  |
| **TB_9** | 1 | 1 | 1 | 1 | 1 | 1 | 1 | 1 | NA |  |  |  |  |  |  |  |  |  |  |  |  |  |  |  |  |  |
| **TB_10** | 1 | 1 | .514 | 1 | 1 | 1 | 1 | 1 | 1 | NA |  |  |  |  |  |  |  |  |  |  |  |  |  |  |  |  |
| **TB_11** | 1 | 1 | 1 | 1 | 1 | 1 | .006 | 1 | .493 | .094 | NA |  |  |  |  |  |  |  |  |  |  |  |  |  |  |  |
| **TB_12** | 1 | 1 | 1 | 1 | 1 | 1 | 1 | 1 | 1 | 1 | 1 | NA |  |  |  |  |  |  |  |  |  |  |  |  |  |  |
| **TB_13** | 1 | 1 | 1 | 1 | 1 | 1 | 1 | 1 | 1 | 1 | 1 | 1 | NA |  |  |  |  |  |  |  |  |  |  |  |  |  |
| **TB_14** | 1 | 1 | 1 | 1 | 1 | 1 | 1 | 1 | 1 | 1 | 1 | 1 | 1 | NA |  |  |  |  |  |  |  |  |  |  |  |  |
| **TB_15** | .390 | .266 | 1 | .040 | .432 | .075 | <.001 | .946 | .017 | .003 | 1 | 1 | 1 | 1 | NA |  |  |  |  |  |  |  |  |  |  |  |
| **TB_16** | 1 | 1 | 1 | 1 | 1 | 1 | 1 | 1 | 1 | 1 | 1 | 1 | 1 | 1 | 1 | NA |  |  |  |  |  |  |  |  |  |  |
| **TB_17** | 1 | 1 | 1 | 1 | 1 | 1 | .066 | 1 | 1 | .398 | 1 | 1 | 1 | 1 | 1 | 1 | NA |  |  |  |  |  |  |  |  |  |
| **TB_18** | 1 | 1 | 1 | 1 | 1 | 1 | 1 | 1 | 1 | 1 | 1 | 1 | 1 | 1 | 1 | 1 | 1 | NA |  |  |  |  |  |  |  |  |
| **TB_19** | 1 | 1 | 1 | 1 | 1 | 1 | 1 | 1 | 1 | 1 | 1 | 1 | 1 | 1 | 1 | 1 | 1 | 1 | NA |  |  |  |  |  |  |  |
| **TB_20** | 1 | 1 | 1 | 1 | 1 | 1 | 1 | 1 | 1 | 1 | 1 | 1 | 1 | 1 | 1 | 1 | 1 | 1 | 1 | NA |  |  |  |  |  |  |
| **TB_21** | 1 | 1 | 1 | 1 | 1 | 1 | 1 | 1 | 1 | 1 | 1 | 1 | 1 | 1 | 1 | 1 | 1 | 1 | 1 | 1 | NA |  |  |  |  |  |
| **TB_22** | 1 | 1 | 1 | 1 | 1 | 1 | 1 | 1 | 1 | 1 | 1 | 1 | 1 | 1 | 1 | 1 | 1 | 1 | 1 | 1 | 1 | NA |  |  |  |  |
| **TB_23** | 1 | 1 | 1 | 1 | 1 | 1 | 1 | 1 | 1 | 1 | 1 | 1 | 1 | 1 | 1 | 1 | 1 | 1 | 1 | 1 | 1 | 1 | NA |  |  |  |
| **TB_24** | 1 | 1 | 1 | 1 | 1 | 1 | 1 | 1 | 1 | 1 | 1 | 1 | 1 | 1 | .294 | 1 | 1 | 1 | 1 | 1 | 1 | 1 | 1 | NA |  |  |
| **TB_25** | 1 | 1 | 1 | 1 | 1 | 1 | 1 | 1 | 1 | 1 | 1 | 1 | 1 | 1 | 1 | 1 | 1 | 1 | 1 | 1 | 1 | 1 | 1 | 1 | NA |  |
| **TB_26** | 1 | 1 | 1 | 1 | 1 | 1 | .322 | 1 | 1 | .945 | 1 | 1 | 1 | 1 | 1 | 1 | 1 | 1 | 1 | 1 | 1 | 1 | 1 | 1 | 1 | NA |

**Appendix 3 table 12**: DP score pairwise comparison - Tier C.

|  | **TC_1** | **TC_2** | **TC_3** | **TC_4** | **TC_5** | **TC_6** | **TC_7** | **TC_8** | **TC_9** | **TC_10** | **TC_11** | **TC_12** | **TC_13** | **TC_14** | **TC_15** | **TC_16** | **TC_17** | **TC_18** | **TC_19** | **TC_20** | **TC_21** | **TC_22** | **TC_23** | **TC_24** | **TC_25** |
| --- | --- | --- | --- | --- | --- | --- | --- | --- | --- | --- | --- | --- | --- | --- | --- | --- | --- | --- | --- | --- | --- | --- | --- | --- | --- |
| **TC_1** | NA |  |  |  |  |  |  |  |  |  |  |  |  |  |  |  |  |  |  |  |  |  |  |  |  |
| **TC_2** | 1 | NA |  |  |  |  |  |  |  |  |  |  |  |  |  |  |  |  |  |  |  |  |  |  |  |
| **TC_3** | 1 | 1 | NA |  |  |  |  |  |  |  |  |  |  |  |  |  |  |  |  |  |  |  |  |  |  |
| **TC_4** | 1 | 1 | 1 | NA |  |  |  |  |  |  |  |  |  |  |  |  |  |  |  |  |  |  |  |  |  |
| **TC_5** | 1 | 1 | 1 | 1 | NA |  |  |  |  |  |  |  |  |  |  |  |  |  |  |  |  |  |  |  |  |
| **TC_6** | 1 | 1 | 1 | 1 | 1 | NA |  |  |  |  |  |  |  |  |  |  |  |  |  |  |  |  |  |  |  |
| **TC_7** | 1 | 1 | 1 | 1 | 1 | 1 | NA |  |  |  |  |  |  |  |  |  |  |  |  |  |  |  |  |  |  |
| **TC_8** | 1 | 1 | 1 | 1 | 1 | 1 | 1 | NA |  |  |  |  |  |  |  |  |  |  |  |  |  |  |  |  |  |
| **TC_9** | 1 | 1 | 1 | 1 | 1 | 1 | 1 | 1 | NA |  |  |  |  |  |  |  |  |  |  |  |  |  |  |  |  |
| **TC_10** | 1 | 1 | 1 | 1 | 1 | 1 | 1 | 1 | 1 | NA |  |  |  |  |  |  |  |  |  |  |  |  |  |  |  |
| **TC_11** | 1 | .304 | .278 | 1 | 1 | 1 | 1 | 1 | 1 | .048 | NA |  |  |  |  |  |  |  |  |  |  |  |  |  |  |
| **TC_12** | 1 | 1 | 1 | 1 | 1 | 1 | 1 | 1 | 1 | 1 | 1 | NA |  |  |  |  |  |  |  |  |  |  |  |  |  |
| **TC_13** | 1 | 1 | 1 | 1 | 1 | 1 | 1 | 1 | 1 | 1 | 1 | 1 | NA |  |  |  |  |  |  |  |  |  |  |  |  |
| **TC_14** | 1 | 1 | 1 | 1 | 1 | 1 | 1 | 1 | 1 | 1 | 1 | 1 | 1 | NA |  |  |  |  |  |  |  |  |  |  |  |
| **TC_15** | 1 | 1 | 1 | 1 | 1 | 1 | 1 | 1 | 1 | 1 | .103 | 1 | 1 | 1 | NA |  |  |  |  |  |  |  |  |  |  |
| **TC_16** | 1 | 1 | 1 | 1 | 1 | 1 | 1 | 1 | 1 | .313 | 1 | 1 | 1 | 1 | .320 | NA |  |  |  |  |  |  |  |  |  |
| **TC_17** | 1 | 1 | 1 | 1 | 1 | 1 | 1 | 1 | 1 | 1 | 1 | 1 | 1 | 1 | 1 | 1 | NA |  |  |  |  |  |  |  |  |
| **TC_18** | 1 | 1 | 1 | 1 | 1 | 1 | 1 | 1 | 1 | 1 | 1 | 1 | 1 | 1 | 1 | 1 | 1 | NA |  |  |  |  |  |  |  |
| **TC_19** | 1 | 1 | 1 | 1 | 1 | 1 | 1 | 1 | 1 | 1 | 1 | 1 | 1 | 1 | 1 | 1 | 1 | 1 | NA |  |  |  |  |  |  |
| **TC_20** | 1 | 1 | 1 | 1 | 1 | 1 | 1 | 1 | 1 | 1 | 1 | 1 | 1 | 1 | 1 | 1 | 1 | 1 | 1 | NA |  |  |  |  |  |
| **TC_21** | 1 | 1 | 1 | 1 | 1 | 1 | 1 | 1 | 1 | 1 | 1 | 1 | 1 | 1 | 1 | 1 | 1 | 1 | 1 | 1 | NA |  |  |  |  |
| **TC_22** | 1 | 1 | 1 | 1 | 1 | 1 | 1 | 1 | 1 | 1 | 1 | 1 | 1 | 1 | 1 | 1 | 1 | 1 | 1 | 1 | 1 | NA |  |  |  |
| **TC_23** | 1 | 1 | 1 | 1 | 1 | 1 | 1 | 1 | 1 | 1 | 1 | 1 | 1 | 1 | 1 | 1 | 1 | 1 | 1 | 1 | 1 | 1 | NA |  |  |
| **TC_24** | 1 | 1 | 1 | 1 | 1 | 1 | 1 | 1 | 1 | 1 | 1 | 1 | 1 | 1 | 1 | 1 | 1 | 1 | 1 | 1 | 1 | 1 | 1 | NA |  |
| **TC_25** | 1 | 1 | 1 | 1 | 1 | 1 | 1 | 1 | 1 | 1 | 1 | 1 | 1 | 1 | 1 | 1 | 1 | 1 | 1 | 1 | 1 | 1 | 1 | 1 | NA |
