# Supplementary material for: Quality of Digital Health Interventions Across Different Health Care Domains: Secondary Data Analysis Study
Source: JMIR Mhealth Uhealth. 2023 Nov 23;11:e47043. doi: 10.2196/47043 (PMC10704310; doi:10.2196/47043)
Supplement: Multimedia Appendix 7 [file mhealth_v11i1e47043_app7.docx]

| **DHI** | **All Rank** | **Tier B Rank** | **Tier C Rank** | **Mean Rank** | **Range** |
| --- | --- | --- | --- | --- | --- |
| Respiratory | 1 | 2 | 2 | 1.6666667 | 1 |
| Urology | 2 | 3 | 1 | 2 | 2 |
| First Aid | 3 | 5 | NA | 4 | 2 |
| Gastrointestinal | 4 | 24 | 4 | 10.666667 | 20 |
| Cardiology | 5 | 15 | 3 | 7.6666667 | 12 |
| Cancer | 6 | 1 | 7 | 4.6666667 | 6 |
| Children’s Health | 7 | 7 | 5 | 6.3333333 | 2 |
| MD | 8 | 13 | 6 | 9 | 7 |
| SSN | 9 | 8 | 25 | 14 | 17 |
| Neurodiverse | 10 | 11 | 21 | 14 | 10 |
| Pregnancy | 11 | 4 | 17 | 10.666667 | 13 |
| Neurological | 12 | 9 | 13 | 11.333333 | 4 |
| UA | 13 | 6 | 14 | 11 | 8 |
| Diabetes | 14 | 12 | 8 | 11.333333 | 4 |
| Dermatology | 15 | 16 | 20 | 17 | 5 |
| PM | 16 | 17 | 11 | 14.666667 | 6 |
| MaCR | 17 | 10 | 15 | 14 | 7 |
| OA | 18 | 14 | 18 | 16.666667 | 4 |
| Healthy Living | 19 | 20 | 10 | 16.333333 | 10 |
| ENTM | 20 | 25 | 9 | 18 | 16 |
| Mental Health | 21 | 18 | 16 | 18.333333 | 5 |
| Dental | 22 | 21 | 22 | 21.666667 | 1 |
| Sexual Health | 23 | 19 | 19 | 20.333333 | 4 |
| Women’s Health | 24 | 22 | 12 | 19.333333 | 10 |
| Allergy | 25 | 23 | 24 | 24 | 2 |
| Ophthalmology | 26 | 26 | 23 | 25 | 3 |

## Appendix 7 – DHI rank consistency

**Appendix 8 table 1:** Rank based on ORCHA score

**Appendix 8 table 2:** Rank based on PCA score

| **DHI** | **All Rank** | **Tier B Rank** | **Tier C Rank** | **Mean Rank** | **Range** |
| --- | --- | --- | --- | --- | --- |
| First Aid | 1 | 1 | NA | 1 | 0 |
| Respiratory | 2 | 2 | 2 | 2 | 0 |
| Urology | 3 | 5 | 1 | 3 | 4 |
| OA | 4 | 4 | 9 | 5.666666667 | 5 |
| Cancer | 5 | 3 | 8 | 5.333333333 | 5 |
| Neurodiverse | 6 | 6 | 19 | 10.33333333 | 13 |
| Cardiology | 7 | 10 | 3 | 6.666666667 | 7 |
| Gastrointestinal | 8 | 24 | 4 | 12 | 20 |
| Children’s Health | 9 | 12 | 5 | 8.666666667 | 7 |
| Neurological | 10 | 7 | 15 | 10.66666667 | 8 |
| UA | 11 | 8 | 13 | 10.66666667 | 5 |
| MD | 12 | 13 | 7 | 10.66666667 | 6 |
| SSN | 13 | 17 | 25 | 18.33333333 | 12 |
| Pregnancy | 14 | 11 | 18 | 14.33333333 | 7 |
| Diabetes | 15 | 16 | 10 | 13.66666667 | 6 |
| PM | 16 | 15 | 12 | 14.33333333 | 4 |
| MaCR | 17 | 9 | 16 | 14 | 8 |
| Dental | 18 | 19 | 21 | 19.33333333 | 3 |
| Healthy Living | 19 | 23 | 11 | 17.66666667 | 12 |
| Dermatology | 20 | 14 | 24 | 19.33333333 | 10 |
| Mental Health | 21 | 22 | 14 | 19 | 8 |
| Allergy | 22 | 20 | 22 | 21.33333333 | 2 |
| ENTM | 23 | 25 | 6 | 18 | 19 |
| Women’s Health | 24 | 21 | 17 | 20.66666667 | 7 |
| Sexual Health | 25 | 18 | 23 | 22 | 7 |
| Ophthalmology | 26 | 26 | 20 | 24 | 6 |

**Appendix 8 table 3:** Rank based on UX score

| **DHI** | **All Rank** | **Tier B Rank** | **Tier C Rank** | **Mean Rank** | **Range** |
| --- | --- | --- | --- | --- | --- |
| Cardiology | 1 | 1 | 6 | 2.666666667 | 5 |
| Diabetes | 2 | 4 | 2 | 2.666666667 | 2 |
| UA | 3 | 5 | 1 | 3 | 4 |
| Cancer | 4 | 3 | 11 | 6 | 8 |
| Allergy | 5 | 9 | 10 | 8 | 5 |
| MD | 6 | 8 | 5 | 6.333333333 | 3 |
| Neurological | 7 | 13 | 4 | 8 | 9 |
| Respiratory | 8 | 10 | 8 | 8.666666667 | 2 |
| PM | 9 | 16 | 3 | 9.333333333 | 13 |
| First Aid | 10 | 18 | NA | 14 | 8 |
| Pregnancy | 11 | 6 | 14 | 10.33333333 | 8 |
| SSN | 12 | 11 | 25 | 16 | 14 |
| Neurodiverse | 13 | 17 | 23 | 17.66666667 | 10 |
| MaCR | 14 | 7 | 15 | 12 | 8 |
| Healthy Living | 15 | 15 | 9 | 13 | 6 |
| Mental Health | 16 | 19 | 17 | 17.33333333 | 3 |
| Urology | 17 | 2 | 21 | 13.33333333 | 19 |
| Children’s Health | 18 | 20 | 19 | 19 | 2 |
| Dermatology | 19 | 12 | 13 | 14.66666667 | 7 |
| OA | 20 | 21 | 24 | 21.66666667 | 4 |
| Dental | 21 | 22 | 22 | 21.66666667 | 1 |
| Gastrointestinal | 22 | 23 | 12 | 19 | 11 |
| ENTM | 23 | 25 | 7 | 18.33333333 | 18 |
| Sexual Health | 24 | 14 | 18 | 18.66666667 | 10 |
| Women’s Health | 25 | 24 | 16 | 21.66666667 | 9 |
| Ophthalmology | 26 | 26 | 20 | 24 | 6 |

**Appendix 8 table 4:** Rank based on DP score

| **DHI** | **All Rank** | **Tier B Rank** | **Tier C Rank** | **Mean Rank** | **Range** |
| --- | --- | --- | --- | --- | --- |
| Gastrointestinal | 1 | 7 | 1 | 3 | 6 |
| Respiratory | 2 | 1 | 7 | 3.3333333 | 6 |
| UA | 3 | 2 | 10 | 5 | 8 |
| Urology | 4 | 4 | 18 | 8.6666667 | 14 |
| Cancer | 5 | 3 | 8 | 5.3333333 | 5 |
| Pregnancy | 6 | 6 | 3 | 5 | 3 |
| Cardiology | 7 | 20 | 5 | 10.666667 | 15 |
| Diabetes | 8 | 16 | 9 | 11 | 8 |
| First Aid | 9 | 5 | NA | 7 | 4 |
| MD | 10 | 9 | 2 | 7 | 8 |
| MaCR | 11 | 8 | 15 | 11.333333 | 7 |
| Women’s Health | 12 | 25 | 4 | 13.666667 | 21 |
| Mental Health | 13 | 11 | 12 | 12 | 2 |
| Sexual Health | 14 | 17 | 6 | 12.333333 | 11 |
| SSN | 15 | 10 | 24 | 16.333333 | 14 |
| Healthy Living | 16 | 13 | 11 | 13.333333 | 5 |
| Children’s Health | 17 | 12 | 16 | 15 | 5 |
| Neurodiverse | 18 | 14 | 25 | 19 | 11 |
| Dental | 19 | 15 | 22 | 18.666667 | 7 |
| Neurological | 20 | 21 | 14 | 18.333333 | 7 |
| PM | 21 | 19 | 13 | 17.666667 | 8 |
| Dermatology | 22 | 18 | 20 | 20 | 4 |
| ENTM | 23 | 24 | 17 | 21.333333 | 7 |
| OA | 24 | 22 | 21 | 22.333333 | 3 |
| Ophthalmology | 25 | 26 | 19 | 23.333333 | 7 |
| Allergy | 26 | 23 | 23 | 24 | 3 |
