# Supplementary material for: Quality of Digital Health Interventions Across Different Health Care Domains: Secondary Data Analysis Study
Source: JMIR Mhealth Uhealth. 2023 Nov 23;11:e47043. doi: 10.2196/47043 (PMC10704310; doi:10.2196/47043)
Supplement: Multimedia Appendix 8 [file mhealth_v11i1e47043_app8.docx]

## Appendix 8 – Distribution of DHIs across NICE ESF tiers by healthcare domain

| **Healthcare domain** |  | **Tier A** | **Tier B** | **Tier C** | **Total** |
| --- | --- | --- | --- | --- | --- |
| **First aid** | Count | 0 | 14 | 0 | 14 |
|  | % within First Aid | 0% | 100% | 0% | 100% |
| **Urology** | Count | 0 | 12 | 3 | 15 |
|  | % within Urology | 0% | 80% | 20% | 100% |
| **Respiratory** | Count | 0 | 49 | 28 | 77 |
|  | % within Respiratory conditions | 0% | 63.6% | 36.4% | 100% |
| **Older adult** | Count | 1 | 10 | 2 | 13 |
|  | % within Caring for Elderly | 7.69% | 76.9% | 15.4% | 100% |
| **Cancer** | Count | 0 | 37 | 17 | 54 |
|  | % within Cancer | 0% | 68.5% | 31.5% | 100% |
| **Cardiology** | Count | 0 | 16 | 18 | 34 |
|  | % within Cardiology | 0% | 47.1% | 52.9% | 100% |
| **Neurological** | Count | 2 | 104 | 30 | 136 |
|  | % within Neurological conditions | 1.47% | 76.5% | 22.1% | 100% |
| **Gastrointestinal** | Count | 0 | 13 | 11 | 24 |
|  | % within Gastrointestinal conditions | 0% | 54.2% | 45.8% | 100% |
| **Neurodiversity** | Count | 1 | 48 | 3 | 52 |
|  | % within Neurodiversity | 1.92% | 92.3% | 5.77% | 100% |
| **Children’s health** | Count | 0 | 66 | 5 | 71 |
|  | % within Child Health | 0% | 93.0% | 7.04% | 100% |
| **Musculoskeletal** | Count | 0 | 30 | 23 | 53 |
|  | % within Musculoskeletal conditions | 0% | 56.6% | 43.4% | 100% |
| **Diabetes** | Count | 0 | 33 | 48 | 81 |
|  | % within Diabetes | 0% | 40.7% | 59.3% | 100% |
| **Utilities or administration** | Count | 0 | 41 | 14 | 55 |
|  | % within Utilities/ Administration | 0% | 74.5% | 25.5% | 100% |
| **Pregnancy** | Count | 0 | 56 | 26 | 82 |
|  | % within Pregnancy | 0% | 68.3% | 31.7% | 100% |
| **Social support network** | Count | 0 | 16 | 1 | 17 |
|  | % within Social support network | 0% | 94.1% | 5.88% | 100% |
| **Dental** | Count | 0 | 20 | 5 | 25 |
|  | % within Dental care | 0% | 80% | 20% | 100% |
| **Medicines and clinical reference** | Count | 4 | 95 | 49 | 148 |
|  | % within Medicines and Clinical Reference | 2.70% | 64.2% | 33.1% | 100% |
| **Pain management** | Count | 0 | 35 | 9 | 44 |
|  | % within Pain Management | 0% | 79.5% | 20.5 | 100% |
| **Ear, Nose, Throat, Mouth** | Count | 0 | 17 | 6 | 23 |
|  | % within Ear/ Nose/ Throat/ Mouth | 0% | 73.9% | 26.1% | 100% |
| **Healthy living** | Count | 6 | 436 | 106 | 548 |
|  | % within Healthy Living | 1.09% | 79.6% | 19.3 | 100% |
| **Dermatology** | Count | 0 | 16 | 13 | 29 |
|  | % within Dermatology | 0% | 55.2% | 44.8% | 100% |
| **Mental health** | Count | 2 | 332 | 102 | 436 |
|  | % within Mental Health | .459% | 76.1% | 23.4% | 100% |
| **Allergy** | Count | 0 | 9 | 5 | 14 |
|  | % within Allergy | 0% | 64.3% | 35.7% | 100% |
| **Women's health** | Count | 0 | 36 | 31 | 67 |
|  | % within Women's Health | 0% | 53.7% | 46.3% | 100% |
| **Sexual health** | Count | 0 | 27 | 31 | 58 |
|  | % within Sexual Health | 0% | 46.6% | 53.4% | 100% |
| **Ophthalmology** | Count | 0 | 30 | 26 | 56 |
|  | % within Ophthalmology | 0% | 53.6% | 46.4% | 100% |
